# Theory-Based Social Media Intervention for Nonmedical Use of Prescription Opioids in Young Adults: Protocol for a Randomized Controlled Trial

**DOI:** 10.2196/65847

**Published:** 2025-03-26

**Authors:** Cheuk Chi Tam, Sean D Young, Sayward Harrison, Xiaoming Li, Alain H Litwin

**Affiliations:** 1 Arnold School of Public Health University of South Carolina Columbia, SC United States; 2 South Carolina SmartState Center for Healthcare Quality University of South Carolina Columbia, SC United States; 3 School of Medicine and Informatics University of California, Irvine Irvine, CA United States; 4 Department of Psychology University of South Carolina Columbia, SC United States; 5 School of Health Research Clemson University Greenville, SC United States; 6 School of Medicine Greenville University of South Carolina Greenville, SC United States; 7 Prisma Health Greenville, SC United States

**Keywords:** nonmedical use of prescription opioids, opioid misuse, young adults, social media, psychosocial intervention, randomized controlled trial, mixed methods

## Abstract

**Background:**

The nonmedical use of prescription opioids (NMUPO) in young adults in the United States is concerning and is robustly influenced by many psychosocial factors. Given the advantages of flexibility, wide coverage, and real-time responses and assessment, using social media appears to be a promising and innovative approach to delivering psychosocial intervention to young adults. However, few theory-based social media interventions are available for NMUPO targeting this at-risk population.

**Objective:**

Guided by the information-motivation-behavioral skills model, the proposed research aims to address critical gaps by theoretically exploring psychosocial content associated with NMUPO among young adults via formative assessment. These findings will then be used to develop and evaluate the feasibility and preliminary efficacy of a peer-led social media intervention to reduce NMUPO among young adults.

**Methods:**

The proposed study will comprise serial research activities. First, formative research will be conducted through semistructured interviews among 30 young adults engaged in NMUPO. Qualitative data will be synthesized using a pragmatic approach for identifying psychosocial content associated with NMUPO. Second, qualitative findings will be used for developing a peer-led social media intervention to reduce NMUPO among young adults by integrating promising psychotherapy principles and incorporating them with well-trained recovery coaches. Third, the social media intervention will be evaluated through a 12-week randomized controlled trial among 70 young adults (n=35, 50% in the intervention group and control group) engaged in NMUPO via mixed methods, including pre- and postintervention surveys, social media paradata (eg, time-series reactions to posts) collection, and ecological momentary assessment during the intervention. The control group will not receive an intervention but will complete the pre- and postintervention surveys. The primary outcomes will be feasibility, usability, and acceptability, while the secondary outcomes will be psychosocial and behavioral measures, such as past–3-month NMUPO, intention, psychological distress, self-efficacy, resilience, and coping strategies.

**Results:**

The proposed study was funded in May 2024. Social media campaigns have received responses from a total of 379 individuals, with 24 (6.3%) identified as eligible. As of February 10, 2025, we have completed formative interviews with 8 eligible participants.

**Conclusions:**

The proposed study will be one of the first efforts to develop and deliver a theory-based peer-led intervention on social media, incorporating empirical findings on the psychosocial mechanism of NMUPO. The findings of the proposed study will provide valuable insights into opioid risk reduction for young adults through an innovative approach. If the tested trial is found to be feasible, the proposed study will contribute to future scaled-up and fully powered psychosocial interventions among young adults and other key populations at risk for NMUPO.

**Trial Registration:**

ClincialTrials.gov NCT06469749; https://clinicaltrials.gov/study/NCT06469749

**International Registered Report Identifier (IRRID):**

DERR1-10.2196/65847

## Introduction

### Background

The nonmedical use of prescription opioids (NMUPO) is a critical public health concern in the United States, and young adults are particularly susceptible to this behavior. NMUPO refers to the aberrant use of prescription opioids in a manner other than as prescribed [1-3]. Substance use literature has paid increasing attention to NMUPO owing to its adverse consequences. National US data have shown a substantial increase of opioid-related overdose deaths, from <5000 in 1999 to >100,000 in 2023 [4,5]. NMUPO has also led to extensive medical costs in the United States (US $78.5 billion annually) [6]. Young adults (aged between 18 and 25 y) are at a high risk for NMUPO. In 2019, 5.5% of young adults in the United States engaged in past-year NMUPO, compared to 2.3% to 3.5% for other age groups. This rate was even higher among those who were not enrolled in college (6.3%) [7]. Besides the risk of overdose and dependence, NMUPO is associated with other negative outcomes, including health-jeopardizing behaviors (eg, driving under the influence), sexual risk behaviors, and suicide attempts [1,8-11]. In response to this issue, many US jurisdictions have launched initiatives to monitor prescription opioids [12]. Despite an obvious reduction in the prescribing rate (eg, 47% in 2019 to 38% in 2023) [12], NMUPO-related deaths remain high (>100,000 in 2023). Notably, NMUPO is strongly linked with the initiation of heroin and synthetic opioid use (eg, fentanyl) in young adults, posing a substantial risk for the development of substance use disorder (SUD) and overdose [13]. Hence, interventions targeting young adults are urgently needed to address NMUPO, and those should be delivered beyond the college population [14].

Interventions for NMUPO in young adults should take psychosocial factors into account. NMUPO literature has identified several important psychosocial factors associated with NMUPO in young adults, including perceived stress (eg, academic strain and traumatic stress), psychiatric distress (eg, depression and anxiety), perception of risk, peer and family influences, and sensation seeking [15-18]. Tam and his colleagues’ previous research on NMUPO in college students has indicated several additional factors that reduce risk for NMUPO, such as psychological resilience, positive coping, outcome expectancies, and self-esteem [10,19-21]. Importantly, psychosocial factors robustly contribute to NMUPO. A meta-analysis study revealed that pooled effect sizes of psychosocial factors on NMUPO (odds ratios 2.14-2.45) were higher than those of somatic symptoms (odds ratio 0.81-1.76), which are traditionally viewed as major NMUPO determinants [22]. These findings indicate that interventions should be guided by a structured framework between psychosocial factors and health actions for NMUPO.

There are a handful of interventions that have been recently developed to address psychosocial aspects of NMUPO in young populations [23-25]. Although these programs have shown some efficacy, they encounter numerous challenges. First, most programs have been adapted from existing substance use interventions that were not originally designed for NMUPO. For example, some content specific to opioids (eg, the risk for overdose and pain relief expectancy) are absent from these programs, yet these factors significantly contribute to health action related to NMUPO. Second, most programs were delivered in schools and failed to target young adults who did not attend college or were difficult to reach. Third, the flexibility of these interventions was limited because they were delivered by professionals in an in-person setting. Furthermore, literature on substance misuse prevention in young adults has revealed several drawbacks to the in-person context, such as limited trust and fear of being stigmatized [26]. These challenges highlight the critical need for an innovative intervention that is based on psychosocial theory, tailored to the NMUPO context, and flexible in real-world settings.

### Psychosocial Theoretical Framework for an NMUPO Intervention

**Figure 1:**
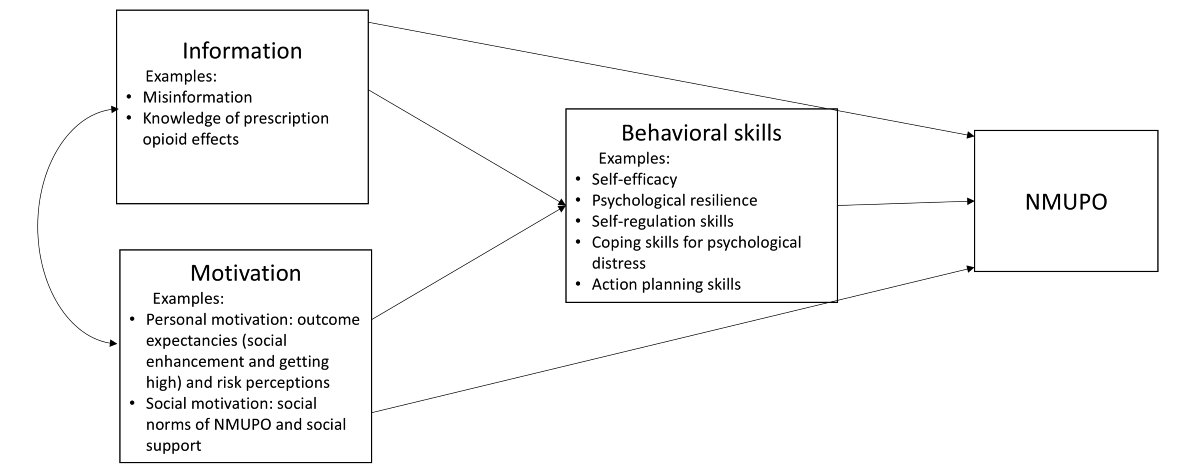
Theoretical framework for the proposed study. NMUPO: nonmedical use of prescription opioids.

To guide an intervention addressing psychosocial aspects of NMUPO, a theoretical framework (Figure 1) was developed based on the information-motivation-behavioral skills (IMB) model [27]. The IMB model has been applauded for its strength in considering a straightforward path diagram for health behavior changes [28]. This model emphasizes that health behavior changes are driven by 3 arrays of psychosocial constructs—information, motivation, and behavioral skills. Information refers to knowledge contributing to a prerequisite for enacting the changes (eg, misinformation of opioids). Motivation includes 2 belief components: personal motivation (eg, outcome expectancies and risk perceptions) and social motivation (eg, social norms and support). Behavioral skills stand for cognitive capacities (eg, self-efficacy and resilience) and behavioral skill sets necessary for adopting the changes (eg, coping or self-regulation skills) or for managing barriers (eg, stress management) [27,28]. Information and motivation can directly affect the target behavior as well as indirectly affect it through the acquisition of adjacent behavioral skills. Such a well-structured framework offers clear guidance for intervention practices and suggests that intervention delivery could benefit from targeting information and motivation factors in the initial sessions, along with enhancing the role of behavioral skill training gradually in the intervention. For instance, initial efforts ought to address misinformation and outcome expectancies of substance use, and, as the intervention progresses, sessions should facilitate action by helping participants develop planning strategies and coping skills. Indeed, existing evidence has shown the efficacy of IMB-guided interventions in reducing substance use (ie, tobacco and illicit drugs) [29-32]. Thus, the IMB model is an ideal theoretical choice for developing an intervention for NMUPO.

### Social Media as a Promising Platform for an NMUPO Intervention Targeting Young Adults

The rapid development of social media technologies provides a novel tool for substance use interventions. Increasing substance use literature has highlighted the significant potential of social media, in which individuals can obtain substance use knowledge, communicate their experiences and thoughts of substance use problems, and seek out social support from peers with similar problems through available networks or groups [33,34]. It is worth noting that social media offers distinct merits in addressing existing challenges in interventions for NMUPO in young adults. First, recent research has validated the use of social media to reach people who engage in NMUPO from multiple venues across the United States [35,36]. Ubiquitous connectivity enables interventions to deliver content remotely to young adults who are not enrolled in college and, therefore, not able to access traditional models of education on safe substance use. Second, social media allows interactive posts in multiple formats (eg, texts, images, polls, and videos) and with customized functions (eg, internet-based goal setting tools and notifications) without time restrictions. This can facilitate exposure to intervention content at the time when participants choose to engage [37]. Third, social media technologies are applicable to timely measurements, such as ecological momentary assessment (EMA) and paradata (eg, auxiliary data of engagement [frequencies of shares, comments, and “likes,” etc] analyses) [38,39], which allow tracking of intervention usability and acceptability on specific modules in real time. Extant literature has shown that EMA has been applied in intervention research for substance use reduction and psychosocial improvements [40]. Fifth, social media is extremely popular among young adults. The latest national data indicate that 72% of US adults use social media, most of whom are young adults (84%) who use it daily (70%) [41].

Another notable feature of social media is that it is significantly favorable for the peer role model, which has been a robust intervention approach for reducing substance use, particularly among young adults [42]. Existing evidence has demonstrated that social media interventions are effective in promoting behavior change for stigmatizing behaviors, including substance use, with perceived peer influence and support playing an important role in the change [43]. Accordingly, emerging peer-led intervention programs have been initiated on popular social media platforms among young adults (ie, Instagram [Meta Platforms, Inc], Facebook [Meta Platforms, Inc], Snapchat [Snap Inc], and Twitter [subsequently rebranded X; X Corp]) and have indicated good feasibility and preliminary efficacy in reducing various substance use behaviors (eg, risky drinking, cigarette use, and cannabis use) [37,42-47]. Notably, promising findings have also been reported for NMUPO reduction. Young et al [45] conducted a pilot study of a peer-led trial on Facebook for patients with chronic pain, who were randomly assigned to the peer-led intervention group or the control group (with no peer leaders) The preliminary results suggested higher engagement and more discussion on NMUPO and coping in the intervention group than in the control group. Taken together, the advantage for the peer role model with other promising aspects, social media appears to be a promising platform for NMUPO intervention among young adults, and such programs would be strongly powered by psychosocial theory–guided content and peer-led modules.

### The Aims of This Study

#### Overview

This study proposes to develop and pilot-test a theory-guided, peer-led social media intervention on Instagram as well as examine its feasibility and preliminary efficacy among young adults engaged in recent NMUPO via a randomized controlled trial (RCT). The assessments will use mixed methods to collect various types of data, including qualitative assessment of semistructured interviews; psychometric measures in pre- and postintervention surveys; real-time paradata on social media platforms; and brief, timely measures in EMA surveys during the intervention. The specific aims are mentioned below.

#### Aim 1

Aim 1 involves conducting a formative study to inform a theory-based social media intervention for NMUPO among young adults in the United States. By collaborating with 4 peer leaders trained as “recovery coaches,” the proposed study will recruit 30 young US adults (aged 18-25 y) who engage in NMUPO from social media (eg, Instagram) and conduct in-depth semistructured interviews to explore psychosocial content associated with NMUPO. The interviews will be guided by the IMB model.

#### Aim 2

Aim 2 involves developing a theory-based social intervention to reduce NMUPO among young adults. The intervention will be developed upon findings from the formative study and based on several promising strategies (eg, peer support group, ambivalence resolving techniques, interactive training modules, internet-based goal setting, and video modeling). It will be delivered via Instagram private groups by peer leaders, with close supervision provided by experts and clinicians in the fields of health psychology and addiction medicine.

#### Aim 3

Aim 3 involves testing the feasibility and preliminary efficacy of the theory-based social media intervention with a randomized controlled design for NMUPO among young adults. Participants who engage in recent NMUPO will be randomly assigned to 1 of the 2 parallel groups, including the intervention group (35/70, 50%) and control group (35/70, 50%), using a 1:1 allocation ratio. The intervention will be evaluated in terms of feasibility, usability, acceptability, engagement, dose, and preliminary efficacy using mixed methods (eg, surveys, EMA, paradata, and semistructured interviews). During the intervention, EMA (a prompt every 2 days) will assess the acceptability and usability of individual modules. Preliminary efficacy will be tested on behavioral outcomes (ie, past-month NMUPO) and psychosocial factors (eg, outcome expectancy, self-efficacy, action or coping planning, resilience, and psychiatric symptoms).

## Methods

### Research Settings and Participants

#### Research Platforms

The proposed formative and intervention study will be conducted on Instagram, which is a popular social media platform for young US adults (71% use) [41,48] and home to many support groups for substance use prevention and reduction [49,50]. Recruitment sites will expand to Snapchat and Facebook (>65% use) [41], which are identified as useful recruitment sources for substance use prevention in young US adults [51,52]. As a pilot study for a cross-platform trial, the module prototypes will be developed based on Instagram features available on other platforms (eg, live streaming, voting polls, group chatting, and multimedia posting). Recent interventions developed on these features indicated efficacy on behavioral changes, including drug use [37,45,46,53-56].

#### Peer Leaders

The study will engage 4 peer leaders who are serving in recovery programs at substance use–related associations or communities for young adults (ie, Gamecock Recovery at the University of South Carolina). The study takes advantage of this model in various aspects, including recruitment and intervention development or delivery. Eligible leaders will be those who are (1) aged between 18 and 25 years, (2) formally trained as a recovery coach (>40 h), (3) have had successful recovery from opioid misuse or opioid use disorder, and (4) use social media (eg, Instagram, Facebook, and Snapchat) on a daily basis. The health psychologist and addiction treatment experts and doctoral-level clinical psychology trainees at the Integrated Care for Recovery (I-CaRe) Training Center at the University of South Carolina will provide training and ongoing support for the peer leaders. e-gift cards will be provided to compensate for their time (US $2 for each recruitment; US $30/h for intervention).

#### Participants and Recruitment

Participants for the formative study and intervention trial will be those who meet the following inclusion criteria: (1) aged between 18 and 25 years, (2) US residents, (3) engaged in NMUPO in the past 3 months, and (4) using Instagram at least 3 times a week in the past 3 months. NMUPO refers to the occurrence of the following behaviors (1 time or more): (1) taking a prescription opioid without a prescription, (2) taking more doses than prescribed, and (3) using opioid for a nonmedical reason (eg, getting high) [1-3]. Individuals will be excluded if they report (1) receiving substance use interventions in the past 3 months, (2) being diagnosed with SUD, or (3) not being proficient in English. We anticipate a total sample size of 100, with 30 for the formative study and 70 for the intervention trial.

**Figure 2:**
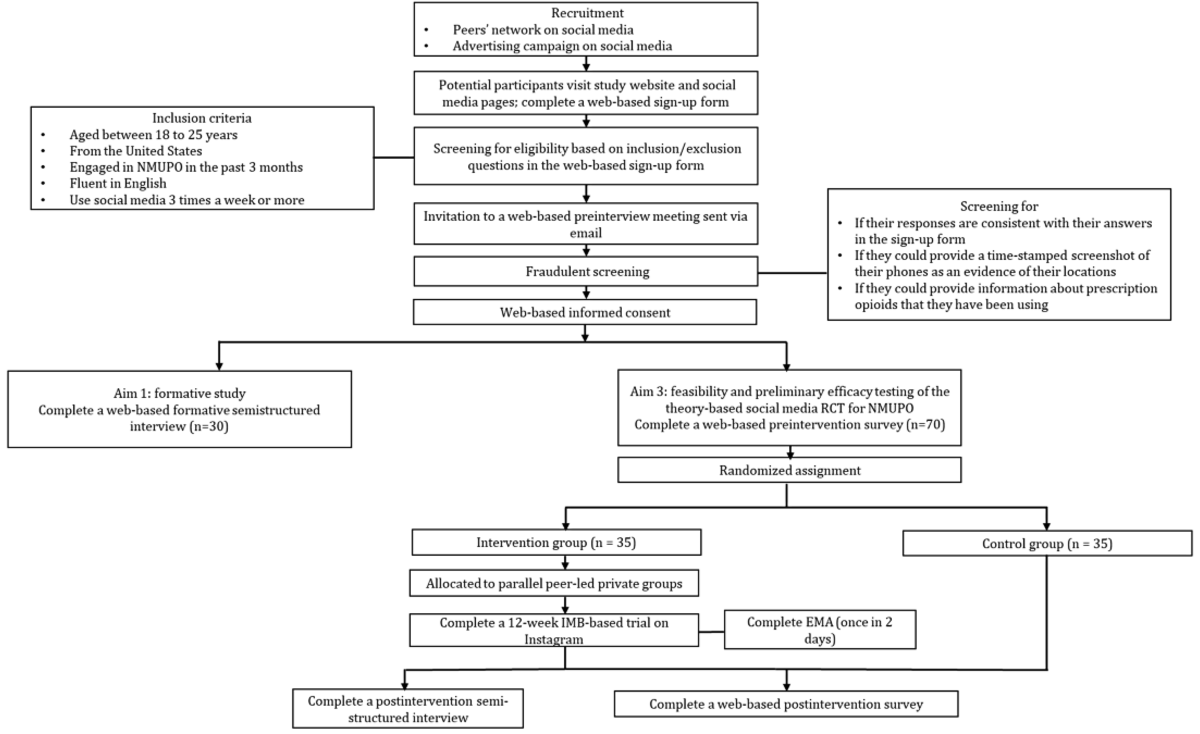
CONSORT (Consolidated Standards of Reporting Trials) flowchart of the proposed study. EMA: ecological momentary assessment; IMB: information-motivation-behavioral skills; NMUPO: nonmedical use of prescription opioids; RCT: randomized controlled trial.

The entire recruitment procedure is presented in Figure 2. Recruitment will be conducted using two strategies: (1) peer outreach and (2) advertising campaigns. For the peer outreach approach, peer leaders will distribute recruitment advertisements via online social networking (eg, posting advertisements on their social media accounts and inviting subscribers to repost the advertisements). Peers can also send direct invitations to young adults who they personally know to be eligible for the study. For advertising campaign approaches, we will target the accounts (on Instagram or Facebook) of colleges, local young adult communities (eg, trade unions), and substance use support groups. Upon approval from their group administrators, we will post advertisements in the groups. In addition, we will develop targeted advertising using Ads Manager (Meta Platforms, Inc) [57]. The advertisements will display research-relevant images, hashtags, and headings, which are tailored to engaging content for young adults. To ensure appropriateness, the advertisements will be reviewed by a community advisory board. The advertisements for aim 3 will be improved in line with formative research. The Ads Manager will also determine the range of the advertisement campaign in terms of age, time, and locations (ie, the United States). The designed advertisements will be delivered in various formats dependent on the platforms, such as linear posts within personal feeds on Instagram, news feeds on Facebook, and stories on Snapchat. To ensure representativeness, recruitment will be stratified based on key demographics (eg, gender, race or ethnicity, and education) in line with the latest US national data on NMUPO [58]. The advertisements will navigate potential participants to a web-based sign-up survey on REDCap (Research Electronic Data Capture; Vanderbilt University), which is a secured web-based platform monitored by the University of South Carolina. The prescreening survey will include questions in terms of age, past–3-month NMUPO (National Institute on Drug Abuse [NIDA]–Modified Alcohol, Smoking and Substance Involvement Screening Test [59]), US residency, use of social media, and SUD diagnosis or treatment history. Participants who are identified as eligible will provide their preferred contact method (eg, Zoom [Zoom Communications, Inc]) in the survey for a brief preinterview online meeting with the study team for a fraudulent screening. Such a screening will be conducted by checking for inconsistencies in their responses, requesting time-stamped screenshots to prove their locations, and asking for information (eg, brand names) of prescription opioids that they have been using. Eligible participants with no fraud responses will then be invited to complete web-based informed consent. Young adults enrolled in the study will be provided with instructions for interviews (aim 1) or intervention (aim 3).

Our recruitment approaches are considered feasible according to existing data. Feasibility research examined the advertising campaign approach on Facebook to reach young adults engaged in NMUPO, showing that it successfully recruited 689 participants over 2 weeks (91% past-year misuse) [35]. The US national data indicate that the majority of young people with NMUPO do not have SUDs (2801/3257, 85.9%) [60]. Previous substance use studies indicated retention rates of 75% to 93% among young adults reached by social media [46,53,61].

### Study Design and Procedures

#### Aim 1: Formative Study

Young adults who are social media users (eg, Instagram and Facebook) and indicate NMUPO will be invited to web-based 60-minute individual semistructured in-depth interviews. The interview guide will be based on the IMB model and aim to (1) identify NMUPO patterns (eg, drug classes and individual behaviors), (2) understand psychosocial factors contributing to reducing NMUPO, (3) extract vivid examples related to psychosocial content, (4) identify planning processes and coping skills facilitating the change, (5) assess the feasibility and acceptability of social media to manage NMUPO, and (6) review recruitment advertisements. This will provide the foundational knowledge and materials to develop specific content (eg, video and posts) for the intervention. The interviewers will be trained research assistants from the fields of psychology or public health. As suggested by the guideline of qualitative research [62], the sample size will be 30 young adults.

Interviews will be audio recorded, transcribed, and coded using a pragmatic approach. The framework analysis [63] will be performed via a deductive process, including five key steps: (1) familiarizing the data, (2) developing a coding scheme, (3) condensing and structuring the data, (4) rearranging the coded data and comparing patterns, and (5) mapping and interpretation [64]. A codebook will be developed based on step 2 as a guide for coding. Coding disagreements will be resolved through group discussion. Data analysis will be conducted on NVivo (version 11.0; Lumivero). Interrater reliability will be assessed throughout the coding process with a goal of ≥0.80 [65].

#### Aim 2: Intervention Development

##### Intervention Strategies

The intervention will apply various principles in psychotherapy. The intervention materials (texts, videos, and images) and activities will be developed using the findings from the formative study. To address particular factors in the IMB model, it will be based on peer support for behavioral change, motivational interviewing (MI) [66], cognitive behavioral therapy (CBT) [67], and solution-focused therapy (SFT) [68], which have been widely applied in substance use interventions [69-71]. Peer support is promising for facilitating social support and skill coaching [72]. MI is used to resolve cognitive ambivalence (eg, positive expectancies of substance use vs the benefits of making change), which is particularly beneficial for promoting motivation [73]. CBT is widely applied to distress management by reframing negative thoughts and facilitating adaptive coping [74]. SFT emphasizes a focus on problem-solving and is favorable for setting concrete goals (action planning) [75,76]. This content and these activities will be packed in different modules.

Incorporating inputs from peer leaders, the video content will follow a video prompting strategy [77]. Videos will feature peer leaders and involve breaking tutorials into steps, allowing young adults to learn and rehearse cognitive and behavioral skills in a sequential fashion. For example, guided by CBT, a peer leader may lead participants may lead participants to reflect on the context, thoughts, and consequences, related to their last experience of NMUPO, and then practice adaptive responses (eg, positive reframing and replacement behaviors). Video prompting has been effective for skill coaching in young adults [78].

##### Intervention Components

###### Overview

**Figure 3:**
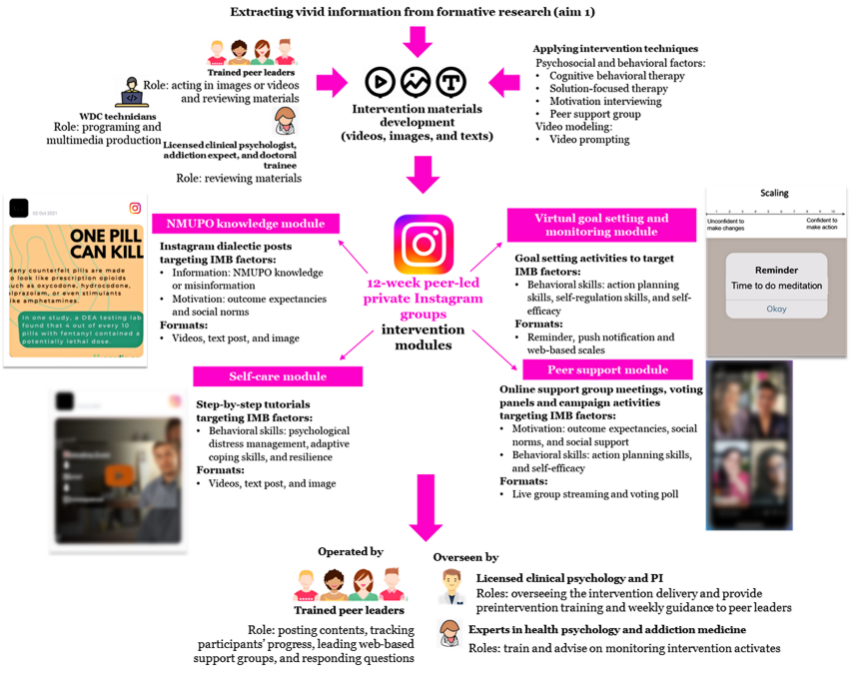
Planned intervention modules and strategies. I-CaRe Training Center: Integrated Care for Recovery Training Center; IMB: information-motivation-behavioral skills; NMUPO: nonmedical use of prescription opioids; PI: principal investigator; WDC: Web Development & Communication.

The proposed intervention components are guided by the IMB-based conceptual framework (Figure 1), with each component targeting specific factors derived from 3 psychosocial constructs (ie, information, motivation, and behavioral skills). As outlined in the framework, the pacing of intervention is structured to prioritize addressing information and motivation factors during the initial phases, while later sessions progressively promote behavioral skill training that contributes to psychological distress management and NMUPO reduction. Incorporating Instagram functions, the intervention platform is proposed to consist of four intervention modules, including (1) NMUPO knowledge module, (2) self-care module, (3) internet-based goal setting or monitoring module, and (4) peer support module (Figure 3). These are planned, and final content will be informed by or modified in line with the formative research. The research team will work closely with the community advisory board and peer leaders to ensure appropriate content and language. The intervention will last 12 weeks according to previous social media trials (typically 8-12 wk) [37,44,79-86]. Clinicians will review the intervention materials and ensure the scope is appropriate. Content will be prompted daily as suggested by the IMB model (eg, initially focus on information and motivation factors and gradually enhance the role of behavioral skills).

###### NMUPO Knowledge Module

A self-paced psychoeducation module will be developed targeting information and motivation factors. This module will provide the latest information about prescription opioids (eg, illicit manufacture and the role of fentanyl, naloxone, and xylazine) and the harms of NMUPO, introduce the benefits of use reduction, and clarify misconceptions. Each component will display vivid stories drawn from the formative study and delivered via multiple formats, including images, videos, and text. Participants can react to the posts by leaving comments or by liking the posts. The majority of the content will be based on MI strategies (eg, ambivalence resolution). This media campaign strategy, combined with prevention education, has been identified as an essential approach for reducing NMUPO in young adults [25].

###### Self-Care Module

This module will provide tutorial materials on behavioral skills for managing psychological distress and identifying strengths to make changes. Material formats (videos, texts, and images), duration, and content will be determined by the findings from the formative study. For example, serial 3-minute tutorial videos will introduce CBT strategies for reframing negative thoughts (eg, “I’m a total failure”) and exploring intrapersonal or interpersonal strengths (eg, resilience). Tutorials will be step-by-step based, allowing participants to role-play and rehearse, presented with vivid examples extracted from the formative research (aim 1). Such a self-guided CBT component has proven effective in reducing psychiatric distress [87].

###### Internet-Based Goal Setting or Monitoring Module

This module aims to enhance action planning skills to reduce NMUPO. Specifically, it will assist young adults with setting personalized goals associated with NMUPO reduction and tracking progress toward meeting the goal. Web-based activities will be developed using SFT strategies. For example, the video will guide using scaling questions, which help participants identify their confidence levels to stop NMUPO and make a realistic plan based on it. Participants will then be instructed to explore barriers and their corresponding coping strategies for moving up the scale. Peer leaders will track their progress and provide support via online support group meetings. In addition, tutorials will instruct participants to set daily reminders or notifications on their devices (eg, mobile phone) to monitor the progress. This virtual self-monitoring has been a promising strategy to manage psychiatric distress and substance use [88].

###### Peer Support Module

This module will provide interpersonal activities for discussing NMUPO, sharing strategies, monitoring progress, providing social support, and increasing self-efficacy to take action, with themes based on the aim 1 findings. Activities include weekly (30 min) online support groups, discussion polls, and campaign activities. Online support groups are live video meetings led by peer leaders and assisted by doctoral-level clinical psychology trainees at the I-CaRe Training Center, who will be overseen by a licensed clinical psychologist and principal investigator (PI), under supervision from experts in health psychology and addiction medicine (SH and AHL). Each meeting will discuss personal practices related to content in that week. Peer leaders will use techniques (eg, active listening, insights, and interpretation) [26] to facilitate cohesion and provide feedback. Discussion polls will be voting activities (eg, selecting adaptive coping skills), aiming to stimulate discussions. Campaign activities will be held weekly with target-orientated themes (eg, “#healthy coping challenge”) to boost posting. Participants will be encouraged to leave comments and peer leaders will promptly provide feedback.

##### Intervention Encouragement and Monitoring

Several strategies that are shown to be useful for enhancing engagement will be used [82,84,89-92]: (1) reminder notifications (via Instagram or emails); (2) prompt replies to participants’ messages; and (3) biweekly prize draws of US $25 gift cards for those who post, attend meetings, and reply on the panels. The intervention will be overseen by PI and a licensed clinical psychologist at the I-CaRe Training Center, under supervision from experts in addiction medicine and psychotherapy for SUD. They will provide preintervention training (eg, skills in MI or CBT techniques and findings from aim 1) and weekly guidance to peer leaders. They will also advise on referrals to link participants to licensed mental and behavioral health professionals in their states of residence if necessary. The doctoral-level psychology trainees at I-CaRe Training Center will support intervention delivery by (1) ensuring conversations are intervention related and banning inappropriate content; (2) promptly replying to participants’ comments; and (3) monitoring participants’ safety (eg, emotional reactions to the posts and in the online group meetings) and offering referrals for outside psychological services, if needed. They will also be responsible for reporting adverse events to the PI if observed.

#### Aim 3: Intervention Feasibility and Preliminary Efficacy Evaluation Study

##### RCT Design

The intervention modules will be tested through an RCT design using mixed methods in 70 young adults who engage in NMUPO [93]. The primary purpose is to determine acceptability, usability, feasibility, and initial efficacy. Participants reached by peer leaders or advertisements on social media will be navigated to a screening survey on REDCap and scheduling a meeting with the research team for informed consent. Participants will be required to comply with the user safety agreement. Participants will be randomly assigned to the parallel intervention (35/70, 50%) or control group (35/70, 50%) using computer-generated random numbers. Participants are blinded to the randomized assignments. Intervention group will be guided to join Instagram peer-led private groups (n=10-15 each). Participants will be asked to provide their Instagram accounts to their assigned peer leaders for participating in intervention activities. Peer leaders will set up private and independent groups via their Instagram accounts and will send direct invitations to their assigned participants to join groups. Once group membership is confirmed, peer leaders will post intervention materials and lead group activities according to the plan as developed in aim 2. Private groups will synchronously deliver intervention packages over 12 weeks. To ensure confidentiality, participants will be assigned unique study IDs, which will be used for data collection and intervention activities. The intervention group will participate in data collection through multiple formats, including Instagram paradata, pre- and postintervention surveys, EMA surveys, and postintervention semistructured interviews.

The control group will not engage in any intervention activities but will complete the pre- and postintervention surveys.

##### Web-Based Pre- and Postintervention Surveys

The web-based pre- and postintervention surveys will be developed and administered on REDCap [94] to measure demographic variables as well as psychosocial and behavioral factors outlined in the IMB-based conceptual framework. Factors include NMUPO behaviors (past–3-month NMUPO) and information- and motivation-related factors (ie, NMUPO knowledge, NMUPO risk perception, outcome expectancies, self-efficacy, psychological distress, resilience, and other substance use). Web-based surveys will be conducted among participants in both the intervention and control groups. Participants will receive survey links via inbox messages to the Instagram accounts they provide for the study. The preintervention survey will be sent 2 weeks before the intervention (baseline), and the postintervention survey will be sent in the week immediately following the intervention (12-week follow-up). The survey will be self-administered and take approximately 20 to 30 minutes to complete. If participants cannot complete the surveys at one time, they can save their responses, and the platform will generate a code that allows them to resume the survey within the same week. The survey will also provide forward and back buttons, allowing participants to review and change their responses before they submit their surveys. Study IDs and filling time will be collected for determining duplicate entries. e-gift cards will be provided for the completion of surveys (baseline and 12 weeks; US $30 each).

##### Use of EMA

EMA will be used through brief evening surveys to screen the prompt acceptability and usability (the Usefulness, Satisfaction, and Ease of use questionnaire) [95] and the dose of particular modules in the intervention group. The EMA survey will be conducted once every 2 days during the intervention. The daily EMA is widely used in behavioral trials [39]. Data will be collected via a 20-second survey on a smartphone app (ExpiWell), which is user-friendly and customizable for EMA development and implementation [96]. A prompt will be sent at the moment for assessment and tailored for the intervention materials in the past 2 days. A reminder will be provided if no response is made in 30 minutes. For safety purposes, if an EMA occurs during an incompatible activity (eg, driving), participants will be instructed to ignore or postpone the prompt.

##### Postintervention Semistructured Interviews

At week 12, participants will join semistructured interviews measuring (1) feasibility, usability, and acceptability and (2) reactions to the content, format, concepts, visual presentation, assessments (survey and EMA) and adaptation to other platforms. The interview guide will be based on mobile health (mHealth) studies [97]. The interview will be audio recorded and take approximately 60 minutes via an online meeting application (eg, Zoom).

### Measures

#### Mixed Methods Measures for Primary Outcomes: Intervention Feasibility Outcomes

Multiple forms of data will be collected within 12 weeks during the intervention for the intervention feasibility evaluation.

##### Feasibility, Acceptability, and Usability

Mixed methods will be used. Acceptability will be assessed using EMA on Likert items that rate how helpful the module is (1=not at all to 5=extremely). Usability will be measured using EMA on the Usefulness, Satisfaction, and Ease of use questionnaire [95], which assesses perceived usefulness, satisfaction, and ease of use for the module. At week 12, participants will join semistructured interviews measuring (1) feasibility, usability, and acceptability and (2) reactions to the content, format, concepts, visual presentation, assessments (survey and EMA), and adaptation to other platforms. The interview guide will be based on mHealth studies [97].

##### Engagement

Paradata on participants’ interactions with specific modules will be collected, including their frequency of reactions (ie, comments, “likes,” questions, and replies on specific posts) and personal posts to Instagram groups [37]. Retention will be assessed by calculating the percentage of participants engaging in the group at varying time points. Data will be collected in real time.

##### Dose of Intervention

Participants’ engagement in particular intervention modules will be measured in EMA surveys as guided by dose operationalization for mHealth interventions [98]. Dose for specific modules will be assessed in three domains (1 yes or no item each): (1) intervention actions (if viewing a post), (2) participant actions (if practicing a skill or completing an assignment), and (3) behavioral target actions (if adopting skills outside of the intervention).

#### Web-Based Surveys for Demographic Variables and Secondary Outcomes: Psychosocial and Behavioral Outcomes

The web-based surveys will measure demographic factors and psychosocial and behavioral factors outlined in the IMB-based conceptual model (Figure 2).

##### Demographic Characteristics

In the baseline preintervention survey, participants will be asked to report their background information regarding gender (ie, man, woman, transgender, or other), race or ethnicity (eg, White or African American or Black), age (y), socioeconomic status, education, family or household characteristics, and physical health status (Patient Health Questionnaire-15) [99].

##### NMUPO Measurement

The past–3-month NMUPO will be measured using relevant items from the Tobacco, Alcohol, Prescription medication, and other Substance use tool [100]. The scale includes 2 sections, with a screener followed by a brief assessment. The screener contains 1 item asking the frequency of NMUPO (ie, used just for feeling, more than prescribed, or without a prescription) with 5 response options (daily or almost daily, weekly, monthly, less than monthly, or never). Participants with a response other than “never” will be led to the brief assessment, including 3 dichotomous questions regarding their level of use, dependence, and concern from others related to the past–3-month NMUPO. The sum score of the brief assessment will be calculated, with a higher score indicating a greater level of NMUPO.

##### NMUPO Knowledge

Information about NMUPO and relevant topics, such as misconceptions, the role of fentanyl, naloxone, and xylazine, will be assessed using the NMUPO knowledge scale [61]. This scale will be developed according to the measure in POP4Teens and the formative study. This scale is proposed to include 15 statements related to opioids or NMUPO. Participants will be asked to determine if a statement is true or not (0=false and 1=true). Responses will then be rated by the research team, with higher scores indicating better knowledge of NMUPO.

##### Outcome Expectancies

Beliefs on positive or negative consequences of engaging in NMUPO will be assessed using a scale adapted from the Behaviors, Expectancies, Attitudes, and College Health Questionnaire [101]. A total of 50 items will be scored on a 5-point Likert-type scale ranging from 0 (not at all) to 4 (very often or always), with higher sum scores standing for the greater level of expectancies. The scale consists of 8 dimensions related to specific expectancies of opioids, including pain reduction, tension reduction, academic preference, emotion enhancement, social enhancement, guilt and dependence, cognitive impairment, and physical discomfort.

##### NMUPO Risk Perception

Perceived susceptibility and severity of engaging in NMUPO will be assessed using the Perceived Risk Scale for Prescription Drug Abuse [102]. This scale includes 5 items with 4 rating options (1=“strongly disagree” and 4=“strongly agree”). The sum score will be generated, with a higher score indicating a greater level of perception risk for NMUPO.

##### Action Self-Efficacy and Coping Self-Efficacy

Perceived control on making actions to stop or reduce NMUPO or confidence on coping with barriers against the actions will be assessed using an adapted version of the Self-Efficacy Scale [103]. A total of 6 items will be rated on a 5-point Likert scale (1=strongly disagree and 5=strongly agree) and can be organized into 2 subscales (coping self-efficacy and action self-efficacy) with 3 items each. The sum scores will be generated for each subscale, with higher scores indicating greater levels of self-efficacy.

##### Psychological Distress

Depression and anxiety symptoms in the past 2 weeks will be assessed using the Patient Health Questionnaire-9 [104] and Generalized Anxiety Disorder-7 [105], respectively. The Patient Health Questionnaire-9 is composed of 9 items asking the frequency of depressive mood experiences (eg, “feeling down, depressed, and hopeless”), while the Generalized Anxiety Disorder-7 comprises 7 items assessing the frequency of feelings of nervousness or worry (eg, “not being able to stop or control worrying”). Two scales have 4 response options ranging from 0 (not at all) to 4 (nearly every day). Sum scores for 2 scales will be generated, with higher scores indicating greater levels of depression or anxiety.

##### Resilience

The Connor-Davidson Resilience Scale will be used for assessing psychological resilience [106]. This scale has 25 items asking about personal capacities in response to stress, including tenacity, tolerance of negative affect, positive acceptance of change, and positive view of adversities. Items will be rated on a 5-point scale (1=not at all and 5=nearly all the time). The sum score will be generated, with a higher score indicating a greater level of resilience.

##### Other Substance Use

The engagement in the use of alcohol, illicit drugs, and cigarettes in the past 3 months will be assessed using the NIDA-Modified Alcohol, Smoking and Substance Involvement Screening Test [59]. The scale includes 12 dichotomous items (0=no and 1=yes) asking if a participant has engaged in using or misusing any of 12 individual substances.

### Data Analysis

The intervention trial will use multiple data analytic methods according to the format of the data.

#### Qualitative Analyses

Postintervention interviews will be audio recorded, transcribed, and coded using a pragmatic approach. The framework analysis [63] will be performed via a deductive process, including five key steps: (1) familiarizing the data, (2) developing a coding scheme, (3) condensing and structuring the data, (4) rearranging the coded data and comparing patterns, and (5) mapping and interpretating [64]. A codebook will be developed based on step 2 as a guide for coding. Coding disagreements will be resolved through group discussion. Data analysis will be conducted by the PI and graduate assistants on NVivo (version 11.0). Interrater reliability will be assessed throughout the coding process with a goal of ≥0.80 [65]. Analyses for postintervention interviews will synthesize the comments on feasibility, acceptability, and usability of the intervention, as well as summarize suggestions for improvements.

#### Quantitative Analyses

##### Bivariate Analyses

Data of web-based surveys (ie, before and after intervention) will be screened for missing data, outliers, and normality. If data are not missing (completely) at random or have a high missing rate (>5%), multiple imputation will be used [107]. Transformations will be conducted if violating normality (square root for moderate skew or log for greater skew). Attrition tests will be used (ie, chi-square tests and ANOVA) on demographics and outcomes at baseline. Descriptive statistics will be reported on primary (feasibility outcomes) and secondary outcomes (psychosocial and behavioral outcomes).

##### Multivariate Analyses

Preliminary outcomes from baseline and 12 week will be tested using repeated measures analysis of covariance, controlling for demographics. EMA data will be tested using multilevel modeling (ie random-coefficient regression model) to examine intervention usability and acceptability, capable of incorporating tests of time-serial characteristics, including linear trend over time and cyclicity. Before multilevel modeling, unconditional means models will be tested for between- and within-participant variation in each measure and determination of appropriate within-person error covariance structure [108].

##### Mixed Method Analysis

Qualitative and quantitative data on feasibility will be triangulated with a complementary proposition [109], in which the qualitative findings will provide in-depth understanding for the quantitative results in terms of intervention feasibility outcomes.

### Power Analysis

The primary aim is to evaluate the feasibility, acceptability, and usability of the intervention to support a future large-scale RCT. According to the guidance for pilot intervention studies [110], it is suggested a minimum sample of 30 per group for the feasibility evaluation. In terms of the aim for the preliminary efficacy evaluation (ie, secondary outcomes of psychosocial and behavioral factors associated with NMUPO), we estimate an effect size according to a previous review on digital interventions for illicit drug use [111] and found small-to-medium effect sizes (Cohen *d*=–0.17 to –0.34) with a 6-month follow-up assessment. G-power analysis [112] estimated a sample size of 10 to 35 per arm for an RCT (repeated measures analysis of covariance). According to previous mHealth trials for substance use with retention rates ranging from 75% to 93% [46,53,61], a sample of 70 appears to be feasible and provides adequate power for proposed analytic plans. Despite a small sample size, this is appropriate for the aim of developmental research.

### Project Timeline

A detailed study timeline is shown in Table 1.

**Table 1:** The project timeline.

		Year 1	Year 2	Year 3	Year 4	Year 5
**Phase 1: formative study (aim 1)**		✓	✓			
	Literature review and assessment development (ie, surveys and semistructured interviews)	✓				
	IRB^a^ submission	✓				
	Peer leader recruitment, participant recruitment and assessment, and community advisory board establishment	✓				
	Transcription and data analysis or extraction		✓			
**Phase 2: intervention development (aim 2)**			✓	✓		
	Development of multimedia files or features based on data from formative research		✓			
	Establishment of IMB^b^-based intervention module packages		✓	✓		
	Development of prototype for social media groups and pages (ie, Instagram [Meta Platforms, Inc])			✓		
	Peer leaders’ and administrators’ training (doctoral clinical psychology trainees)			✓		
**Phase 3: intervention feasibility and preliminary efficacy evaluation study (aim 3)**				✓	✓	
	Participant recruitment, preintervention assessment, and randomized controlled trial assignment			✓		
	Delivery of intervention			✓		
	EMA^c^ surveys and paradata collection			✓		
	Postintervention assessment and interviews				✓	
	Data analysis				✓	
	Revisions based on participants’ feedback				✓	
	Dissemination and disclosure of research findings				✓	✓
	Preparation and submission a research proposal for a large-scale intervention effectiveness evaluation with a randomized controlled trial design				✓	✓

^a^IRB: institutional review board.

^b^IMB: information-motivation-behavioral skills.

^c^EMA: ecological momentary assessment.

### Ethical Considerations

All data (ie, qualitative and quantitative) and materials (including video, images, and comments) collected or generated in the proposed study are deidentified and stored on a password-protected drive to ensure confidentiality. Intervention materials will be only delivered in private Instagram groups, which are not accessible to public users. Before their participation, all participants are provided with informed consent that explains the study’s purposes, voluntary nature, and confidentiality. Participants are allowed to withdraw from the study at any time. Participants will receive e-gift cards for the completion of formative interviews (US $50) and each intervention assessment (preintervention and postintervention; US $30 each). The prorated incentive will be given according to the completion of EMA during the intervention period (eg, US $25 e-gift card for a 100% completion; US $15 for a 67% completion). Biweekly random prize draws of US $25 gift cards for these who post, attend meetings, and reply on the panels. At the end of the intervention, US $60 e-gift cards will be provided to participants who complete 90% of tasks during the intervention.

As a multiphase study, the protocol is reviewed by the research ethics boards in a sequential manner. A protocol for a formative interview study (aim 1) has been submitted to relevant institutional boards for review. Detailed information on the ethical approval and recruitment progress is provided in the subsequent sections. The protocol has been developed and reviewed in accordance with the SPIRIT (Standard Protocol Items: Recommendations for Intervention Trials checklist; Multimedia Appendix 1). The protocol of the formative study (aim 1) has been approved by the Institutional Review Board for Human Research at the University of South Carolina (#Pro00135306).

## Results

**Figure 4:**
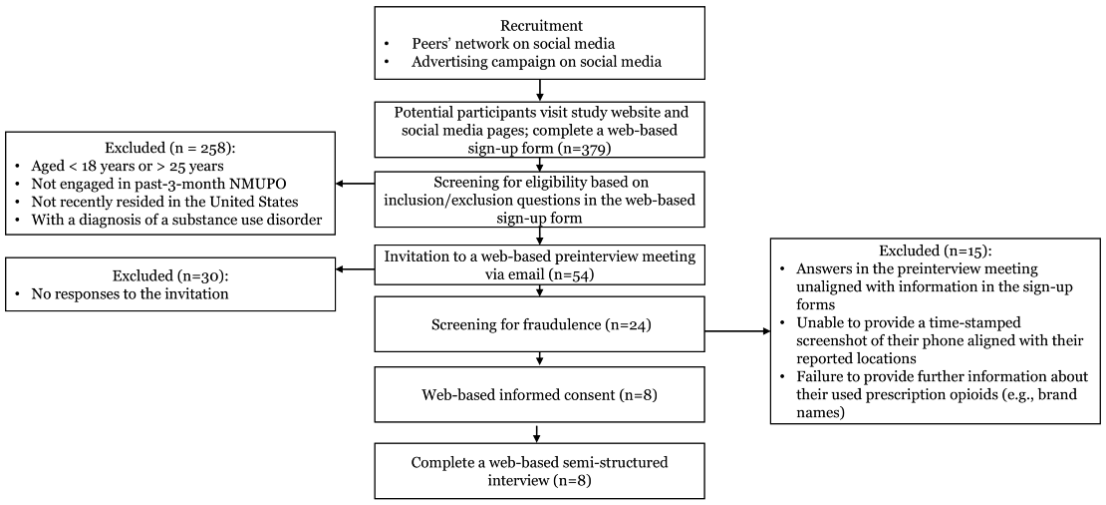
Recruitment progress for the formative study (aim 1) as of February 10. NMUPO: nonmedical use of prescription opioids.

The protocol was funded in May 2024 by the NIDA under the award number 1K01DA058768-01A1. The research team has developed a social media campaign (eg, Facebook and Instagram) to facilitate recruitment for formative qualitative interviews. As of February 10, 2025, the study has received 379 responses, with 24 (6.3%) completing screening interviews. In total, 8 participants who are identified as eligible for the study have consented and completed formative interviews (Figure 4).

Formative interviews (aim 1) are expected to be completed by May 2025. The completion of formative qualitative analyses and intervention module development (aim 2) is anticipated by May 2027. A protocol for an intervention study (aim 3) will be submitted to the Institute Review Board for Human Research for review in early 2027. Upon receipt of an approval, the recruitment of the RCT will be initiated in late 2027, and the intervention will be conducted in early 2028. The intervention feasibility and preliminary efficacy (aim 3) will be finalized by the middle of 2028. Data analyses and manuscript development of the intervention will be completed by the end of 2029.

## Discussion

### Anticipated Findings

In response to the growing evidence on psychosocial aspects of opioid addiction and relevant disorder concerns in the United States, this proposed study aims to apply a psychosocial theoretical framework and peer role model techniques to develop and implement an evidence-based intervention for reducing NMUPO risk in young US adults. The intervention will be established through a serial research activity, from a formative semistructured in-depth interview study to feasibility evaluation of a 12-week interactive peer-led trial. By leveraging the merits of social media, the proposed intervention study will be powered by adopting innovative approaches, including a tailored recruitment strategy (eg, paid advertisement campaigns), intervention delivery that transcends time and space constraints, and the application of mixed methods and time-series assessments (eg, paradata and EMA measures). As one of the first attempts to deliver peer-led interventions on social media specifically targeting NMUPO, the findings from this mixed methods design will offer valuable insights into the use of innovative approaches for reducing opioid risk among young adults. In line with previous intervention studies for NMUPO on social media [45], we anticipate that our intervention will yield high feasibility outcomes. In particular, the mixed methods assessments for the intervention group will demonstrate (1) high feasibility, acceptability, and usability on all intervention modules among young adults across 12 weeks and (2) high engagement, especially for the modules in the interactive formats, such as discussion polls and live streaming group meetings. We also anticipate significant preliminary intervention efficacy on the NMUPO-related and psychosocial behavior outcomes. Compared to the control group, the intervention will report (1) decreases in past–3-month NMUPO and other substance use (eg, alcohol, illicit drugs, and cigarettes), (2) decreases in psychological distress, (3) changes in outcome expectancies (eg, increased physical discomfort and decreased pain reduction), (4) increases in NMUPO knowledge and NMUPO risk perception, and (5) increases in resilience and self-efficacy (ie, action and coping).

Notably, the proposed intervention will be developed and implemented based on qualitative findings on the psychosocial mechanism of NMUPO, incorporating tailored psychotherapy strategies, such as CBT, MI, and peer role models. As highlighted in recent research, NMUPO in young adults is robustly driven by psychosocial factors, with influences comparable to biological and medical factors, which are traditionally viewed as prominent determinants [22]. However, scant theory- and evidence-based intervention programs have been designed to empower psychosocial skills for young US adults, especially for those who have engaged in NMUPO but do not develop addiction or SUDs. As a pilot trial, our feasibility evaluation will contribute to emerging practices of opioid use disorder prevention within social media settings, particularly through the implementation of peer-led groups [45,46]. In addition, preliminary efficacy on psychosocial measures will contribute to the growing literature on psychosocial aspects of NMUPO and offer clinical evidence for using psychosocial intervention to manage the risk of opioid misuse in the young population. The success of the proposed trial should underscore the potential of leveraging social media as a feasible tool for delivering secondary prevention programs targeted at group-based psychosocial skill training for young people at high risk of SUD.

The proposed study has several methodological limitations. First, the generalization of our findings will be limited by the convenient sampling approach. Second, because most evaluations in this study will be based on self-reported measures, results could be subject to response bias (eg, social desirability and recall bias). Third, our EMAs could impose additional burdens on participants due to the frequent prompts for assessments in real time. Fourth, our efficacy findings could be limited in assessing long-term effects given a pre- and postevaluation design. Fifth, since the planned content for all peer-led groups will be identical and delivered concurrently, our analyses could not examine the efficacy of specific intervention modules. Sixth, given a program will be delivered on Instagram, our findings will be limited to Instagram users, and the format and presentation of the intervention content will be constrained by the platform’s available features and functionality. Seventh, our peer leaders could also have limitations. They are recruited solely from local agencies within a single state (South Carolina). In addition, although they will be supported by clinicians (eg, licensed clinical psychologists and physicians in addiction medicine), their service may be subject to disparities in the quality of support. To address those, our future plan is to conduct a scaled-up cross-platform social media RCT study with a design of the multiphase optimization strategy and longitudinal measures. The intervention will be provided by a team of peer leaders, comprising recovery coaches with expertise in opioid misuse from various states across the United States.

The proposed intervention might encounter several potential challenges related to social media, including inefficient recruitment (eg, scams) and intervention delivery affected by the policy changes to social media platforms. Several contingency plans will be used if these occur. To address recruitment challenges, the eligibility of participants will be thoroughly evaluated by a 2-step approach. Young adults who have signed up for participation via social media will be navigated to complete a screening survey regarding their age, current NMUPO, history of SUDs, and their preferred online meeting methods. The research team will then contact the participants by their preferred methods and evaluate their eligibility in person. In addition, the recruitment plan will be supplemented by additional in-person approaches, including a snowball strategy (ie, allowing participants to invite eligible peers), and an outreach approach (ie, advertising at local young adult communities, colleges, and associations). To address the policy challenges to social media, the research team will transfer multimedia files to a publicly available but password-secured platform (eg, Discord [Discord Inc]), which also allows member reactions, and implement all interactive activities (eg, live streaming) via alternative social media applications (eg, WhatsApp [Meta Platforms]).

Guided by a theoretical framework, the proposed intervention study is dedicated to reducing NMUPO behaviors and the risk for opioid-related addiction and disorders by empowering psychosocial competencies among young adults via the application of social media. The proposed program is informed by substantial literature on NMUPO in young adults and formative qualitative findings of psychosocial mechanisms associated with NMUPO behavior management. Findings from rigorous methods are anticipated to inform a future scaled-up RCT study to optimize intervention content and effectiveness of a fully powered cross-platform social media intervention.

### Study Dissemination

To promote the academic and social benefits of the proposed intervention study, several strategies will be used to disseminate the study findings. First, the findings will be published in international and national scientific journals and presented at national and international scientific meetings or conferences held by SUD research institutes. Second, the access to the deidentified data will be available for registered users through our selected data repository, the Interuniversity Consortium for Political and Social Research, with high security standards. Third, documentation and support materials regarding the intervention will be available at ClinicalTrials.gov and compatible with its protocol registration data elements. Fourth, we will capitalize on social media and professional networks that can increase the research and accessibility of findings, such as webinars, files, and videos available on websites and publicly available channels (eg, YouTube [Google LLC]), to increase the visibility and impact of the scientific publications and presentations. We hope that the anticipated success of the proposed intervention will prompt policy attempts for prescription drug intervention and treatments. The lessons learned from the proposed study and the intervention strategies tested can be scaled up to reduce NMUPO risk in young adults and other high-risk populations.

## Appendix

Multimedia Appendix 1 SPIRIT (Standard Protocol Items: Recommendations for Intervention Trials) checklist for the proposed study.

Multimedia Appendix 2 Peer-review report from the IPTA–Interventions to Prevent and Treat Addictions Study Section, National Institute on Drug Abuse (National Institutes of Health, USA).

Multimedia Appendix 3 CONSORT-eHEALTH checklist (V 1.6.1).

## Abbreviations

CBT: cognitive behavioral therapy
EMA: ecological momentary assessment
I-CaRe: Integrated Care for Recovery
IMB: information-motivation-behavioral skills
mHealth: mobile health
MI: motivational interviewing
NIDA: National Institute on Drug Abuse
NMUPO: nonmedical use of prescription opioids
PI: principal investigator
RCT: randomized controlled trial
REDCap: Research Electronic Data Capture
SFT: solution-focused therapy
SPIRIT: Standard Protocol Items: Recommendations for Intervention Trials
SUD: substance use disorder

## Data Availability

The (deidentified) datasets generated or analyzed during this study will be available in the Data Sharing for Demographic Research data repository hosted at the Interuniversity Consortium Political and Social Research.
