# Supplementary material for: Theory-Based Social Media Intervention for Nonmedical Use of Prescription Opioids in Young Adults: Protocol for a Randomized Controlled Trial
Source: JMIR Res Protoc. 2025 Mar 26;14:e65847. doi: 10.2196/65847 (PMC11982775; doi:10.2196/65847)
Supplement: Multimedia Appendix 2 [file resprot_v14i1e65847_app2.pdf]

SUMMARY STATEMENT

(Privileged Communication)

PROGRAM CONTACT:
ALEXA Romberg
301-435-0264
alexa.romberg@nih.gov

Release Date: 10/31/2023
Revised Date:

Application Number: 1K01DA058768-01A1
Formerly: 1K01DA058768-01

Principal Investigator

TAM, CHEUK CHI

Applicant Organization: UNIVERSITY OF SOUTH CAROLINA AT COLUMBIA

Review Group: IPTA
Interventions to Prevent and Treat Addictions Study Section

Meeting Date: 10/12/2023
Council: JAN 2024
Requested Start: 05/01/2024

Opportunity Number: PA-20-176
PCC: CV/ARR

Project Title: Theory-based social media intervention for non-medical use of prescription opioids (NMUPO) in young adults
SRG Action: Impact Score:24 Percentile:7 +
Next Steps: Visit https://grants.nih.gov/grants/next_steps.htm
Human Subjects: 48-At time of award, restrictions will apply
Animal Subjects: 10-No live vertebrate animals involved for competing appl.
Gender: 1A-Both genders, scientifically acceptable
Minority: 1A-Minorities and non-minorities, scientifically acceptable
Age: 7A-Only Adults, scientifically acceptable

Project Year	Direct Costs Requested	Estimated Total Cost
1	141,847	152,243
2	145,856	156,545
3	146,729	157,482
4	146,712	157,464
5	146,454	157,187
TOTAL	727,598	780,922

ADMINISTRATIVE BUDGET NOTE: The budget shown is the requested budget and has not been adjusted to reflect any recommendations made by reviewers. If an award is planned, the costs will be calculated by Institute grants management staff based on the recommendations outlined below in the COMMITTEE BUDGET RECOMMENDATIONS section.

1K01DA058768-01A1 Tam, Cheuk Chi

RESUME AND SUMMARY OF DISCUSSION: This application seeks support for Dr. Cheuk Chi Tam to become an independent researcher with the scientific career focused on examination of psychosocial factors impacting non-medical use of prescription opioids (NUMPO) in young adults. This is a very strong and productive applicant, who is supported by strong letters of recommendation and demonstrates a strong track record of research productivity and publications. The mentoring team is very strong, with relevant and complementary expertise, and with a history of successful mentoring, although some members of the panel noted insufficient expertise in ecological momentary assessment (EMA) represented on the team. The institutional environment is strong and supportive, offering an excellent intellectual environment for the proposed work. The panel noted this resubmission to be responsive to the concerns raised in the prior review. The career development plan was considered comprehensive and well justified and appropriately addressed the weaknesses identified in prior critique. Training in the responsible conduct of research was considered appropriate. The proposed research was considered important, and highly relevant to the applicant's career objectives, with the ability to advance what is currently known about addressing psychosocial factors associated with NOMA among young adults. Some weaknesses in the research plan were noted around the controls and outcome measures, as well as due to the unclear feasibility and high burden of EMA, and limited justification for the use of peer leaders. Concerns were raised around the highly complex research design, with multiple tasks to be accomplished requiring complex analytic skills, and whether the proposed training would be sufficient to accomplish such a complicated project; as well as around insufficient clarity if the candidate has the necessary behavioral intervention background and skills to lead the proposed study. Concerns around human subject protections were also raised due to the insufficiently addressed ethical issues of using peer leaders to deliver the intervention to their peers. Following the discussion, the weaknesses identified were considered addressable and the panel agreed that proposed training and research experiences will provide the applicant with an excellent foundation for a future career to conduct research to reduce NMUPO among young adults.

DESCRIPTION (provided by applicant): Non-medical use of prescription opioids (NMUPO) is a timely and significant public health issue in the United States (US). Young adults are the key population vulnerable to NMUPO. Existing literature indicates that NMUPO in young adults is influenced by a robust array of psychosocial factors. Tailored interventions guided by a psychosocial theory, such as the information-motivation-behavioral skills (IMB) model, are urgently needed for addressing NMUPO in young adults. One innovative approach is to reach out to and deliver psychosocial interventions to young adults via social media technology, yet limited efforts have sought to develop such interventions for NMUPO among young adults. Thus the scientific objective of this K01 is to gather data via IMB-guided formative study regarding psychosocial influences on NMPUO and then to use these findings to inform the development of a peer-led social media intervention designed to reduce NMUPO among young adults. The career development objective of this K01 is to obtain intensive mentored training essential to improving the theoretical and practical expertise needed to develop innovative and implementable peer-led psychosocial interventions for NMUPO in high-risk populations. The training will be supported by the rigorous and highly productive research environment at the University of South Carolina and by a mentor team with expertise in addiction treatment, health psychology, peer support group, formative assessment, social media and psychosocial intervention design, mixed methodology, ecological momentary assessment, as well as intervention development, implementation, and evaluation. Training progression is linked to three specific research aims. In Aim 1, we conduct formative research, guided by the IMB model, to explore psychosocial contents associated with NMUPO in young adults. We then use the findings from Aim 1 to develop a peer-led social media intervention to reduce NMUPO among young adults (Aim 2) by integrating promising psychotherapy principles and incorporating with peer leaders who are well-trained for recovery coaching. In Aim 3, we

conduct a pretesting of the feasibility, acceptability, usability, and preliminary efficacy on NMUPO reduction and psychosocial changes of the theory-based social media intervention with a 12-week pilot randomized controlled trial among 70 NMUPO engaging young adults (35 for intervention and 35 for control) via pre-test, 12-week ecological momentary assessment (during the intervention), and post-tests (12-month follow-ups). This project will contribute to future largescale and fully-powered psychosocial interventions for NMUPO among young adults or other high-risk populations using innovative technology that can address challenges in traditional substance use interventions.

PUBLIC HEALTH RELEVANCE: Non-medical of prescription opioids (NMUPO) in young adults is particularly concerning and is robustly influenced by psychosocial factors. Given the advantages of flexibility, wide coverage, and real-time responses/assessment, social media can be a promising and innovative approach to deliver psychosocial intervention to young adults but yet few theory-based social media interventions are available for NMUPO in this at-risk population. The proposed plan for research and career development addresses the critical gaps by theoretically exploring psychosocial contents associated with NMUPO among young adults via the formative assessment and using these findings to develop and pilot-test the feasibility and preliminary efficacy of a theory-based and peer-led social media intervention to reduce NMUPO and address psychosocial factors among young adults.

CRITIQUE 1

Candidate: 1
Career Development Plan/Career Goals /Plan to Provide Mentoring: 2
Research Plan: 4
Mentor(s), Co-Mentor(s), Consultant(s), Collaborator(s): 1
Environment and Institutional Commitment to the Candidate: 1

Overall Impact: This is a K01 resubmitted application by a productive early career scientist who is proposing to conduct a formative study to inform a theory-based social media intervention to reduce NMUPO among young adults in the US, develop a theory-based social media intervention to reduce NMUPO among young adults, and test feasibility and primary efficacy of the theory-based social media intervention with a randomized controlled design for NMUPO among young adults. The application is largely responsive to prior critiques. The candidate is a PhD prepared developmental psychologist with a concentration in health psychology. The candidate has also completed a postdoc in public health and a fellowship in Fellowship in big data and infectious disease research. Currently employed as a research assistant professor. Candidate has training in intervention development and has collaborated on multiple research grants. Prior work has focused primarily on HIV-related risk behaviors. Has a total of 36 publications, with 14 as first author. The candidate is seeking this K01 to continue growth and development with the following goals: Improve theoretical/practical expertise in formative research and developing psychosocial interventions for peer-led NMUPO in US young adults; Gain theoretical knowledge and practical skills for developing social media interventions; Enhance expertise in implementing and analyzing acceptability, usability, and feasibility of social media intervention using mixed methods and EMA; and Obtain expertise in designing and evaluating a full-powered social media RCT. The career plan is comprehensive and well justified and addresses the weaknesses identified in prior critique, including clarifications in primary mentorship. The mentoring team is strong with a history of successful mentoring. The research strategy addressed an important issue of NMUPO among young adults 18-25 years old and is innovative in its use of social media as the delivery platform for the intervention. Aim 1 and 2 involves a formative study to develop a peer led social media intervention by utilizing in-depth semi-structured interviews. In aim 3, they test the feasibility and primary efficacy of the intervention on past month NMUPO (primary outcome) and self-efficacy, coping, resilience, and psychiatric symptoms (secondary outcomes). The peer led intervention is delivered via Instagram

groups with 10 participants per group. It will incorporate tenets of CBT, MI, and solution focused therapy. The intervention will have four modules – knowledge, selfcare, virtual goal setting, and peer support module. There are some self-paced modules and some weekly synchronous 30-minute online support groups. This will be a 12-week intervention. Daily EMA will be conducted on feasibility, acceptability, usability, and dose). However, some issues in the approach still raise a few questions. It is not clear whether the educational modules (particularly for the intervention group) will be completed asynchronously or synchronously. In some places, the investigators mention that these will be self-paced modules. However, the EMA is also timed to coincide with specific content in evaluating its usability and acceptability. Additionally, if the modules are self-paced, when will the intervention end? This is not clear. Also, it is not clear whether a wait list control will be utilized (per the statements in the introduction but this is not apparent in the research plan). These inconsistencies are quite concerning. The environment is strong and very supportive of the candidate. The study will also use peer leaders to deliver the intervention.

1. Candidate:

Strengths

- Adequately qualified early career investigator with some pilot work that informs this direction of research.
- Has history of collaborating with mentors and demonstrates good working relationships with mentors on the team.
- Able to develop an independent program of research that is complementary to those of mentors.

Weaknesses

- No significant weaknesses identified.

2. Career Development Plan/Career Goals & Objectives:

Strengths

- Training and mentoring goals are adequate and justified.
- There is a specific mentor(s) with relevant expertise to help the candidate achieve each goal.
- The timeline for the training and mentorship is appropriate.

Weaknesses

- No significant weaknesses identified.

3. Research Plan:

Strengths

- The sampling and recruitment procedures are clear.
- Use of validated and theory-driven methods for intervention development.
- Use of validated instruments where applicable.
- The research plan addresses significant issue of NMUPO.
- Leveraging the utility of social media to conduct intention research for young adults.
- The examination of the feasibility, acceptability, and usability of the intervention is well articulated.

- The analytic plans are well justified.

Weaknesses

- It is not clear whether the educational modules (particularly for the intervention group) will be completed asynchronously or synchronously. In some places, the investigators mention that these will be self-paced modules. However, the EMA is also timed to coincide with specific content in evaluating its usability and acceptability.
- If the modules are self-paced, when will the intervention end? This is not clear.
- Also, it is not clear whether a wait list control will be utilized (per the statements in the introduction but this is not apparent in the research plan).

4. Mentor(s), Co-Mentor(s), Consultant(s), Collaborator(s):

Strengths

- All mentors are highly productive scientists with relevant experience.
- The mentors have a history of collaboration with the candidate.
- History of successful mentoring relationship with other junior scientists.
- The expertise of the mentors is complementary to allow the candidate to achieve stated goals and training objectives.

Weaknesses

- No significant weaknesses noted.

5. Environment and Institutional Commitment to the Candidate:

Strengths

- The environment is appropriate to conduct the prosed study.

Weaknesses

- No significant weaknesses noted.

Study Timeline:

Strengths

- The revised timeline is appropriate.

Weaknesses

- No significant weaknesses identified.

Protections for Human Subjects:

Acceptable Risks and Adequate Protections

- Acceptable.

Data and Safety Monitoring Plan (Applicable for Clinical Trials Only):

Acceptable.

- o Acceptable.

Inclusion Plans:

- Sex/Gender: Distribution justified scientifically
- Race/Ethnicity: Distribution justified scientifically
- Inclusion/Exclusion Based on Age: Distribution justified scientifically
- Acceptable.

Vertebrate Animals:
Not Applicable (No Vertebrate Animals)

Biohazards:
Not Applicable (No Biohazards)

Resubmission:

- Yes - application is largely responsive to prior reviews.

Training in the Responsible Conduct of Research:
Acceptable
Comments on Format (Required):

- Acceptable.

Comments on Subject Matter (Required):

- Acceptable.

Comments on Faculty Participation (Required; not applicable for mid- and senior-career awards):

- Acceptable.

Comments on Duration (Required):

- Acceptable.

Comments on Frequency (Required):

- Acceptable.

Select Agents:
Not Applicable (No Select Agents)

Resource Sharing Plans:
Acceptable

Authentication of Key Biological and/or Chemical Resources:
Not Applicable (No Relevant Resources)

Budget and Period of Support:

Recommend as Requested

CRITIQUE 2

Candidate: 1
Career Development Plan/Career Goals /Plan to Provide Mentoring: 2
Research Plan: 4
Mentor(s), Co-Mentor(s), Consultant(s), Collaborator(s): 1
Environment and Institutional Commitment to the Candidate: 1

Overall Impact: This is a K01 proposal for the training of a promising Research Assistant Professor, Dr. Tam. The PI proposes to conduct a 12-week pilot randomized controlled trial (N=70) among young adults with non-medical use of prescription opioids. Training aims center around: improving expertise in formative research and intervention development, training in designing social media interventions, training in mixed methods and EMA research, and training toward social media RCT. The mentorship team is strong, including content experts and those with clinical expertise. The candidate, Dr. Tam, has a strong record of publications, initial success of grant funding, and comes highly recommended by his colleagues. Primary weakness of the proposal center around Aim 3 of the research plan. For example, concern about the feasibility of daily (or twice daily) EMA over a 12 week period, especially given the modest planned renumeration, and lack of clarity on the need for such intensive assessment to determine acceptability and usability outcomes. Second, lack of clarity around the intended and appropriate primary outcomes (e.g., acceptability and usability vs. efficacy). In summary, this is a strong application, the strengths are considerable with just a couple moderate weaknesses in the research plan which are addressable.

1. Candidate:

Strengths

- Candidate has a track record of publication commensurate with his training and years of experience (36 publications, 14 first author.)
- Track record of work in the areas of NMUPO (8 publications.)
- Awarded internal funding to do work in NMUPO.
- Strong letters of recommendations.

Weaknesses

- None noted by reviewer.

2. Career Development Plan/Career Goals & Objectives:

Strengths

- Career development plan clearly aligns with research goals.
- Clear and well developed timeline and overview of planned training activities to achieve training goals.

- Training goals include improving expertise in formative research and intervention development, training in designing social media interventions, training in mixed methods and EMA research, and training toward social media RCT.

Weaknesses

- Minor discrepancies between mentor letter and training plan (e.g., biweekly vs. weekly meetings with primary mentor.)

3. Research Plan:

Strengths

- Mixed methods approach.
- Plans for programming and LOS from Consultant Yang to support development.
- Detailed recruitment plan including mentor studies using similar recruitment approaches successfully.

Weaknesses

- Aim 3 states RCT will focus on feasibility and primary efficacy – yet pilot studies are not intended to assess efficacy, and if the trial were indeed powered for efficacy, then it would mean there is no need for the planned full scale trial proposed subsequent to this project.
- Unclear if EMA is once or twice daily, but regardless, 12 weeks of once or twice daily EMA is highly likely to result in considerable missing data (e.g., exceeding 50%), given evidence of precipitous digital intervention disengagement after 30 days.
- EMA may be unduly burdensome given the data to be collected appears to be acceptability and usability information that may be gathered through less intensive assessment approach.
- NMUPO is not likely to be an appropriate primary outcome given Aim 3 is a pilot trial and focus appears to be on acceptability and usability, though in some instances a primary outcome around behavior change is provided.

4. Mentor(s), Co-Mentor(s), Consultant(s), Collaborator(s):

Strengths

- Dr. Litwin is the primary mentor and will be involved with clinical training, stakeholder engagement, intervention design/delivery, implementation, and supervision of peer support groups, result dissemination, and R01 preparation.
- Dr. Litwin will meeting with Dr. Tam biweekly.
- Dr. Li, Dr. Young, and Dr. Harrison provide complementary expertise as members of the co-mentorship team, with Dr. Li and Dr. Harrison being co-located with Dr. Tam at USC.
- Dr. Yang provides letter of support as a consultant to assist in EMA programming.

Weaknesses

- None noted by reviewer.

5. Environment and Institutional Commitment to the Candidate:

Strengths

- USC School of Public Health provides a strong environment for the training and research proposed.
- Evidence of institutional support in that the PI is already in a Research Assistant Professor role.

Weaknesses

- None noted by reviewer.

Study Timeline:

Strengths

- Training and research timeline are well thought through and appear ambitious but there are contingency plans in place if planned activities take longer than expected.

Weaknesses

- None noted by reviewer.

Protections for Human Subjects:

Acceptable Risks and Adequate Protections

- Adequate protections for human participants.

Data and Safety Monitoring Plan (Applicable for Clinical Trials Only):

Acceptable

- DSMP is provided.

Inclusion Plans:

- Sex/Gender: Distribution justified scientifically
- Race/Ethnicity: Distribution justified scientifically
- Inclusion/Exclusion Based on Age: Distribution justified scientifically
- Research plan includes both sexes between 18-25 years of age, including racial and ethnic minoritized individuals.

Vertebrate Animals:

Not Applicable (No Vertebrate Animals)

Biohazards:

Not Applicable (No Biohazards)

Resubmission:

- Dr. Tam and their mentorship team provide detailed accounting of how they addressed prior critiques. Substantive changes included clarification of potential weaknesses such as clarifying that there are licensed providers on the team, and changes to the study design, including adding a waitlist control arm, adding peer-led components to the intervention, and dropping the focus on stimulants and removing the multi-phase optimization strategy, to now only focus on opioids.

Training in the Responsible Conduct of Research:

Acceptable

Comments on Format (Required):

- Contains both in person and virtual trainings, spanning training types including workshops, coursework, meetings, etc.

Comments on Subject Matter (Required):

- Spans multiple content areas including ethical principles of research, research misconduct, etc.

Comments on Faculty Participation (Required; not applicable for mid- and senior-career awards):

- Several faulty will participate in training, including mentors.

Comments on Duration (Required):

- Training spans the 5 year period.

Comments on Frequency (Required):

- Range from 1 hour/month to 1 semester, and as needed.

Select Agents:

Not Applicable (No Select Agents)

Resource Sharing Plans:

Not Applicable (No Relevant Resources)

Authentication of Key Biological and/or Chemical Resources:

Not Applicable (No Relevant Resources)

Budget and Period of Support:

Recommend as Requested

Recommended budget modifications or possible overlap identified:

- None noted.

CRITIQUE 3

Candidate: 2
Career Development Plan/Career Goals /Plan to Provide Mentoring: 3
Research Plan: 5
Mentor(s), Co-Mentor(s), Consultant(s), Collaborator(s): 1
Environment and Institutional Commitment to the Candidate: 1

Overall Impact: This proposed study addresses an important and timely public health issue – to reduce non-medical use of prescription opioids among young adults using a psychosocial intervention

on social media. The research plan is to understand psychosocial contents associated with NMUPO and information that could inform the intervention using qualitative methods, develop this intervention, and test it with an RCT. Career development plans include gaining theoretical and practical experience in formative research and peer-led psychosocial training for NMUPO in US young adults, gaining knowledge in designing social media interventions, implementing and analyzing mixed methods studies that involve social media and EMA, and obtaining expertise in designing and evaluation social media RCT for substance misuse. Dr. Tam has a strong potential to be a successful independent researcher. He has a great mentoring team who has relevant research experiences and the scientific environment at USC is appropriate. There is some concern that Dr. Tam can lead this ambitious intervention study without sufficient degree/training in this area. The proposed research to develop an intervention to help young adults quit non-medical use of prescription opioids is an important public health goal. The aims are theoretically sound and logically build on each other. The proposed intervention to utilize social media as a delivery platform is also innovative and a strength. A scoring driving weakness is the use of peer leaders. The scientific justification for the use of peer leaders is not provided; it's unclear whether these peer leaders are sufficiently trained to deliver this intensive and complicated intervention without appropriate training, and the ethical issues of using peer leaders to deliver an intervention to their peers are not adequately explained.

1. Candidate:

Strengths

- Dr. Tam has a strong and diverse research background in conducting psychosocial intervention research among various at-risk populations. Dr. Tam has also conducted focused studies to understand psychological influences on NMUPO. These combined experiences provide him with a strong knowledge base to pursue further training in current proposed areas. His research experience and publication records show great promise for an independent research career to conduct research to reduce NMUPO among young adults.

Weaknesses

- Despite the enthusiasm about the candidate and even with the training that Dr. Tam is proposing to receive, it is somewhat less convincing that Dr. Tam who does not have a clinical degree is suitable to lead such an ambitious project. E.g., Dr. Tam will train staff on empathy – unclear what credentials and training he has to deliver this training.

2. Career Development Plan/Career Goals & Objectives:

Strengths

- The didactics effectively address the training areas where Dr. Tam finds gap.

Weaknesses

- Several seminars are proposed. It doesn't seem realistic that the mentors are creating seminars specifically to support Dr. Tam on these various topic areas.
- Too many didactics are proposed in addition to shadowing and clinical training – the topics include the main topic areas and areas related to ethics in the form of bi-weekly seminars (it's unclear whether each topic proposed covers just one class or several classes), and formal courses (n=4). The workload of all this is difficult to determine. Some could be a semester long and some could be short workshops.

3. Research Plan:

Strengths

- The proposed intervention is a comprehensive and ambitious treatment grounded in a strong theoretical foundation, comprising multiple innovative and appealing components. The content areas such as knowledge, self-care, virtual goal setting, and peer support modules delivered in a popular social media platform will help support young adults to quit.
- The content of the intervention is well thought out.
- The three aims are logically connected and build on each other.

Weaknesses

- The score-driving weakness is the use of peer leaders. There is insufficient justification for using peer leaders and there are practical issues with using peer leaders to deliver an intensive intervention such as the one proposed in this application. The issues are: 1) Potential pitfalls with peer leaders recruiting within their own network. For instance, peer leaders can have their friends in a group that they are leading. This may influence the group dynamics, interfere with treatment delivery and effects, and violate confidentiality. 2) The intervention proposed is a complex and intensive intervention for peer leaders to deliver. They require MI approaches and CBT skills and they are going to be asked to assess psychiatric symptoms, which are skills that require extensive training. Although there are plans for supervision by doctoral students – it's unclear whether peer leaders can manage this case load and whether the training will be sufficient. 3) The doctoral students will step in if there is inappropriate content discussed during the intervention. It's unclear how this would play out during the session. This seems quite intrusive and may undermine peer leaders if doctoral students step in to maintain order. 4) Video modeling is done by peer leaders. I assume that the benefit of having peer leaders do video modeling is that participants see their peers modeling behavior. However, there's no guarantee that the peer models featured in the video will be the leader of their intervention so they might not be aware that their peers are modeling behaviors. Additionally, modeling is as good as how well the behaviors are modeled so it may not work out well if not acted properly.
- $2 to recruit an individual is very low and may be insufficient to motivate young adults to recruit.
- The frequency and duration of participant engagement with the intervention components over the 12 week is unclear.

4. Mentor(s), Co-Mentor(s), Consultant(s), Collaborator(s):

Strengths

- Dr. Tam has put together a strong mentoring team with unique and relevant research experiences.
- Dr. Li will mentor on psychosocial interventions and RCTs. He has mentored many successful mentees.
- Dr. Young is an expert in conducting interventions on social media.
- Dr. Litwin's expertise is providing treatments to individuals with opioid use disorders and has a strong record of mentoring early career researchers.

Weaknesses

- None.

5. Environment and Institutional Commitment to the Candidate:

Strengths

- The Department of Health Promotion, Education and Behavior at the University of South Carolina provides strong intellectual and physical support to Dr. Tam to achieve his research and career goals. The faculty and mentors in this department have a strong record of successful research funding and outstanding accomplishments. Dr. Tam is a full time research assistant professor in this department and has the needed support from his department.

Weaknesses

- None.

Study Timeline:

Strengths

- The timeline for training and research is appropriate.

Weaknesses

- None.

Protections for Human Subjects:

Unacceptable Risks and/or Inadequate Protections

- If a peer leader is recruiting participants for the RCT component, there is a possibility that participants in the intervention may be in the same treatment group as their friends/acquaintances and their confidentiality may be violated (i.e., use status). There is no consideration of this breach of confidentiality.

Data and Safety Monitoring Plan (Applicable for Clinical Trials Only):

Acceptable

Inclusion Plans:

- Sex/Gender: Distribution justified scientifically
- Race/Ethnicity: Distribution justified scientifically
- Inclusion/Exclusion Based on Age: Distribution justified scientifically
- The breakdown of sex/gender, race/ethnicity, and age is appropriate. This study is focused on young adults who use NMUPO.

Vertebrate Animals:

Not Applicable (No Vertebrate Animals)

Biohazards:

Not Applicable (No Biohazards)

Resubmission:

- Dr. Tam has adequately addressed most of the reviewers' comments. However, I still have concerns about Dr. Tam's qualifications to lead the behavioral intervention study. The

clarification/addition that the peers will lead the behavioral intervention and be featured in video modeling raises issues (please see comments in the Approach section).

Training in the Responsible Conduct of Research:

Acceptable

Comments on Format (Required):

- The format is appropriate - there is a mix of formal courses, seminars, and online training.

Comments on Subject Matter (Required):

- The subject matter is appropriate; topics include ethics in public health, drug abuse prevention, and social media. However, given that this study is providing psychosocial intervention, there could be more ethical training on clinical intervention.

Comments on Faculty Participation (Required; not applicable for mid- and senior-career awards):

- The strength is that some of the mentors are leading courses that are proposed in the training plan. However, some of their role in other online courses is unclear (e.g., CITI training, guided studies). They are listed as 'faculty' but their role in these trainings is not provided.

Comments on Duration (Required):

- The duration is appropriate.

Comments on Frequency (Required):

- The frequency is appropriate.

Select Agents:

Not Applicable (No Select Agents)

Resource Sharing Plans:

Not Applicable (No Relevant Resources)

Authentication of Key Biological and/or Chemical Resources:

Not Applicable (No Relevant Resources)

Budget and Period of Support:

Budget Modifications Recommended (in amount/time)

Recommended budget modifications or possible overlap identified:

- $4300 for one computer seems high.

THE FOLLOWING SECTIONS WERE PREPARED BY THE SCIENTIFIC REVIEW OFFICER TO SUMMARIZE THE OUTCOME OF DISCUSSIONS OF THE REVIEW COMMITTEE, OR REVIEWERS' WRITTEN CRITIQUES, ON THE FOLLOWING ISSUES:

PROTECTION OF HUMAN SUBJECTS: UNACCEPTABLE. PROTECTION OF HUMAN SUBJECTS: UNACCEPTABLE. The protection of human subjects from research risks is unacceptable. There are concerns about confidentiality protections. This needs to be addressed.

INCLUSION OF WOMEN PLAN: ACCEPTABLE

INCLUSION OF MINORITIES PLAN: ACCEPTABLE

INCLUSION ACROSS THE LIFESPAN: ACCEPTABLE

COMMITTEE BUDGET RECOMMENDATIONS: The budget was recommended as requested.

Footnotes for 1K01DA058768-01A1; PI Name: Tam, Cheuk Chi

+ Derived from the range of percentile values calculated for the study section that reviewed this application.

NIH has modified its policy regarding the receipt of resubmissions (amended applications).See Guide Notice NOT-OD-18-197 at https://grants.nih.gov/grants/guide/notice-files/NOT-OD-18-197.html. The impact/priority score is calculated after discussion of an application by averaging the overall scores (1-9) given by all voting reviewers on the committee and multiplying by 10. The criterion scores are submitted prior to the meeting by the individual reviewers assigned to an application, and are not discussed specifically at the review meeting or calculated into the overall impact score. Some applications also receive a percentile ranking. For details on the review process, see http://grants.nih.gov/grants/peer_review_process.htm#scoring.

MEETING ROSTER

Interventions to Prevent and Treat Addictions Study Section
Risk, Prevention and Health Behavior Integrated Review Group
CENTER FOR SCIENTIFIC REVIEW
IPTA
10/12/2023 - 10/13/2023

Notice of NIH Policy to All Applicants: Meeting rosters are provided for information purposes only. Applicant investigators and institutional officials must not communicate directly with study section members about an application before or after the review. Failure to observe this policy will create a serious breach of integrity in the peer review process, and may lead to actions outlined in NOT-OD-22-044 at https://grants.nih.gov/grants/guide/notice-files/NOT-OD-22-044.html, including removal of the application from immediate review.

CHAIRPERSON(S)

PIPER, MEGAN E, PHD
PROFESSOR
DEPARTMENT OF MEDICINE
UNIVERSITY OF WISCONSIN-MADISON
MADISON, WI 53711

MEMBERS

AROUT, CAROLINE A, PHD *
ASSISTANT PROFESSOR
DEPARTMENT OF PSYCHIATRY
COLUMBIA UNIVERSITY MEDICAL CENTER
NEW YORK, NY 10032

AUDRAIN-MCGOVERN, JANET, PHD
PROFESSOR AND DIRECTOR
DEPARTMENT OF PSYCHIATRY
PERELMAN SCHOOL OF MEDICINE
UNIVERSITY OF PENNSYLVANIA
PHILADELPHIA, PA 19104

BICKEL, WARREN K, PHD *
PROFESSOR
DEPARTMENT OF PSYCHOLOGY
VIRGINIA TECH CARILION RESEARCH INSTITUTE
VIRGINIA POLYTECHNIC INSTITUTE
AND STATE UNIVERSITY
ROANOKE, VA 24016

BINSWANGER, INGRID A, MD
SENIOR CLINICIAN INVESTIGATOR
INSTITUTE FOR HEALTH RESEARCH
KAISER PERMANENTE
AURORA, CO 80014

BRICKER, JONATHAN B, PHD
PROFESSOR
DIVISION OF PUBLIC HEALTH SCIENCES
FRED HUTCHINSON CANCER RESEARCH CENTER
UNIVERSITY OF WASHINGTON
SEATTLE, WA 98109

CHENEY, MARSHALL, PHD *
ASSOCIATE PROFESSOR
DEPARTMENT OF HEALTH AND EXERCISE SCIENCE
UNIVERSITY OF OKLAHOMA
NORMAN, OK 73019

CLAUS, ERIC D, PHD
ASSOCIATE PROFESSOR
DEPARTMENT OF BIOBEHAVIORAL HEALTH
THE PENNSYLVANIA STATE UNIVERSITY
UNIVERSITY PARK, PA 16802

COUGHLIN, LARA NICOLE, PHD *
ASSISTANT PROFESSOR
DEPARTMENT OF PSYCHIATRY
U-M ADDICTION CENTER
UNIVERSITY OF MICHIGAN
ANN ARBOR, MI 48109

COULTER, ROBERT W.S, PHD *
ASSISTANT PROFESSOR
BEHAVIORAL AND COMMUNITY HEALTH SCIENCES
GRADUATE SCHOOL OF PUBLIC HEALTH
PEDIATRICS, SCHOOL OF MEDICINE
UNIVERSITY OF PITTSBURGH
PITTSBURGH, PA 15261

CRUM, KATHLEEN I, PHD *
ASSISTANT PROFESSOR
DEPARTMENT OF PSYCHIATRY
INDIANA UNIVERSITY SCHOOL OF MEDICINE
INDIANAPOLIS, IN 46202

EDWARDS, KATIE M, PHD *
ASSOCIATE PROFESSOR
DEPARTMENT OF EDUCATIONAL PSYCHOLOGY
COLLEGE OF EDUCATION AND HUMAN SCIENCES
UNIVERSITY OF NEBRASKA-LINCOLN
LINCOLN, NE 68583

GAINER, DANIELLE, MD *
ASSOCIATE PROFESSOR
DEPARTMENT OF PSYCHIATRY
WRIGHT STATE UNIVERSITY
DAYTON, OH 45435

KIANG, MATHEW VINHHOA, DSC *
ASSISTANT PROFESSOR
DEPARTMENT OF EPIDEMIOLOGY AND POPULATION HEALTH
STANFORD UNIVERSITY
STANFORD, CA 94305

KONG, GRACE, PHD
ASSOCIATE PROFESSOR
DEPARTMENT OF PSYCHIATRY
YALE UNIVERSITY SCHOOL OF MEDICINE
NEW HAVEN, CT 06519

KOTLYAR, MICHAEL, PHMD *
ASSOCIATE PROFESSOR
DEPARTMENT OF EXPERIMENTAL AND CLINICAL PHARMACOLOGY
COLLEGE OF PHARMACY
UNIVERSITY OF MINNESOTA
MINNEAPOLIS, MN 55455

LABRIE, JOSEPH W, PHD
PROFESSOR
DEPARTMENT OF PSYCHOLOGY
LOYOLA MARYMOUNT UNIVERSITY
LOS ANGELES, CA 90045

LEDGERWOOD, DAVID M, PHD
PROFESSOR
DEPARTMENT OF PSYCHIATRY
AND BEHAVIORAL NEUROSCIENCES
SCHOOL OF MEDICINE
WAYNE STATE UNIVERSITY
DETROIT, MI 48201

MCHUGH, REBECCA KATHRYN, PHD
ASSOCIATE PROFESSOR
DEPARTMENT OF PSYCHIATRY
HARVARD MEDICAL SCHOOL
BELMONT, MA 02478

MCNEELY, JENNIFER, MD
ASSOCIATE PROFESSOR
DEPARTMENTS OF POPULATION HEALTH AND MEDICINE
GROSSMAN SCHOOL OF MEDICINE
NEW YORK UNIVERSITY
NEW YORK, NY 10016

MCPHERSON, STERLING M, PHD
DIRECTOR AND PROFESSOR
PROGRAM OF EXCELLENCE IN ADDICTIONS RESEARCH
DEPARTMENT OF COMMUNITY AND BEHAVIORAL HEALTH
ELSON S. FLOYD COLLEGE OF MEDICINE
WASHINGTON STATE UNIVERSITY
SPOKANE, WA 99210

MONTGOMERY, LATRICE, PHD
DIRECTOR OF CLINICAL RESEARCH AT RIA HEALTH,
ADJUNCT ASSOCIATE PROFESSOR
DEPARTMENT OF PSYCHIATRY AND BEHAVIORAL NEUROSCIENCE
COLLEGE OF MEDICINE
UNIVERSITY OF CINCINNATI
CINCINNATI, OH 45229

MUMBA, MERCY N, PHD
ASSOCIATE PROFESSOR
CAPSTONE COLLEGE OF NURSING
THE UNIVERSITY OF ALABAMA
TUSCALOOSA, AL 35401

PANG, RAINA, PHD *
ASSOCIATE PROFESSOR
DEPARTMENT OF RESEARCH POPULATION AND PUBLIC HEALTH SCIENCES
KECK SCHOOL OF MEDICINE
UNIVERSITY OF SOUTHERN CALIFORNIA
LOS ANGELES, CA 90033

SADASIVAM, RAJANI, PHD
PROFESSOR
DIVISION OF HEALTH AND IMPLEMENTATION SCIENCE
DEPARTMENT OF POPULATION AND QUANTITATIVE HEALTH SCIENCES
UNIVERSITY OF MASSACHUSETTS MEDICAL SCHOOL
WORCESTER, MA 01605

SCHMITZ, JOY MARIE, PHD
PROFESSOR
DEPARTMENT OF PSYCHIATRY AND BEHAVIORAL SCIENCES
UNIVERSITY OF TEXAS HEALTH SCIENCE CENTER
HOUSTON, TX 77054

SINGH, PRAMIL NAND, DRPH *
DIRECTOR AND PROFESSOR
TRANSDISCIPLINARY TOBACCO RESEARCH PROGRAM
SCHOOL OF MEDICINE
CANCER CENTER
LOMA LINDA UNIVERSITY
LOMA LINDA, CA 92350

SPEARS, CLAIRE ADAMS, PHD
ASSOCIATE PROFESSOR
DEPARTMENT OF HEALTH POLICY & BEHAVIORAL SCIENCES
SCHOOL OF PUBLIC HEALTH
GEORGIA STATE UNIVERSITY
ATLANTA, GA 30303

SPILLANE, NICHEA SOLOMON, PHD
ASSOCIATE PROFESSOR
DEPARTMENT OF PSYCHOLOGY
COLLEGE OF HEALTH SCIENCES
UNIVERSITY OF RHODE ISLAND
KINGSTON, RI 02881

STEVENS-WATKINS, DANELLE J, PHD
PROFESSOR
DEPARTMENT OF EDUCATIONAL, SCHOOL, AND
COUNSELING PSYCHOLOGY
COLLEGE OF EDUCATION
UNIVERSITY OF KENTUCKY
LEXINGTON, KY 40506

STORMSHAK, ELIZABETH A, PHD
PROFESSOR
COLLEGE OF EDUCATION
PREVENTION SCIENCE INSTITUTE
UNIVERSITY OF OREGON
EUGENE, OR 97403

VILARDAGA, ROGER, PHD
ASSOCIATE PROFESSOR
DEPARTMENT OF PSYCHIATRY AND BEHAVIORAL
SCIENCES
SCHOOL OF MEDICINE
DUKE UNIVERSITY
DURHAM, NC 27710

WALLEY, ALEXANDER YALE, MD *
PROFESSOR
GENERAL INTERNAL MEDICINE
BOSTON UNIVERSITY SCHOOL OF MEDICINE
BOSTON, MA 02118

YI, RICHARD, PHD
PROFESSOR AND DIRECTOR
COFRIN LOGAN CENTER FOR ADDICTION RESEARCH
AND TREATMENT
DEPARTMENT OF PSYCHOLOGY
UNIVERSITY OF KANSAS
LAWRENCE, KS 66045

SCIENTIFIC REVIEW OFFICER

ZANDBERG, IZABELLA, PHD
SCIENTIFIC REVIEW OFFICER
CENTER FOR SCIENTIFIC REVIEW
BETHESDA, MD 20892

EXTRAMURAL SUPPORT ASSISTANT

AMARE, MERON ERMIAS
LEAD EXTRAMURAL SUPPORT ASSISTANT
CENTER FOR SCIENTIFIC REVIEW
NATIONAL INSTITUTES OF HEALTH
BETHESDA, MD 20892

* Temporary Member. For grant applications, temporary members may participate in the entire meeting or may review only selected applications as needed.

Consultants are required to absent themselves from the room during the review of any application if their presence would constitute or appear to constitute a conflict of interest.
