# Supplementary material for: Theory-Based Social Media Intervention for Nonmedical Use of Prescription Opioids in Young Adults: Protocol for a Randomized Controlled Trial
Source: JMIR Res Protoc. 2025 Mar 26;14:e65847. doi: 10.2196/65847 (PMC11982775; doi:10.2196/65847)
Supplement: Multimedia Appendix 3 [file resprot_v14i1e65847_app3.pdf]

CONSORT-EHEALTH (V 1.6.1) - Submission/Publication Form

The CONSORT-EHEALTH checklist is intended for authors of randomized trials evaluating web-based and Internet-based applications/interventions, including mobile interventions, electronic games (incl multiplayer games), social media, certain telehealth applications, and other interactive and/or networked electronic applications. Some of the items (e.g. all subitems under item 5 - description of the intervention) may also be applicable for other study designs.

The goal of the CONSORT EHEALTH checklist and guideline is to be
a) a guide for reporting for authors of RCTs,
b) to form a basis for appraisal of an ehealth trial (in terms of validity)

CONSORT-EHEALTH items/subitems are MANDATORY reporting items for studies published in the Journal of Medical Internet Research and other journals / scientific societies endorsing the checklist.

Items numbered 1., 2., 3., 4a., 4b etc are original CONSORT or CONSORT-NPT (non-pharmacologic treatment) items.
Items with Roman numerals (i., ii, iii, iv etc.) are CONSORT-EHEALTH extensions/clarifications.

As the CONSORT-EHEALTH checklist is still considered in a formative stage, we would ask that you also RATE ON A SCALE OF 1-5 how important/useful you feel each item is FOR THE PURPOSE OF THE CHECKLIST and reporting guideline (optional).

Mandatory reporting items are marked with a red *.
In the textboxes, either copy & paste the relevant sections from your manuscript into this form - please include any quotes from your manuscript in QUOTATION MARKS,
or answer directly by providing additional information not in the manuscript, or elaborating on why the item was not relevant for this study.

YOUR ANSWERS WILL BE PUBLISHED AS A SUPPLEMENTARY FILE TO YOUR PUBLICATION IN JMIR AND ARE CONSIDERED PART OF YOUR PUBLICATION (IF ACCEPTED).
Please fill in these questions diligently. Information will not be copyedited, so please use proper spelling and grammar, use correct capitalization, and avoid abbreviations.

DO NOT FORGET TO SAVE AS PDF _AND_ CLICK THE SUBMIT BUTTON SO YOUR ANSWERS ARE IN OUR DATABASE !!!

Citation Suggestion (if you append the pdf as Appendix we suggest to cite this paper in the caption):
Eysenbach G, CONSORT-EHEALTH Group
CONSORT-EHEALTH: Improving and Standardizing Evaluation Reports of Web-based and Mobile Health Interventions
J Med Internet Res 2011;13(4):e126
URL: http://www.jmir.org/2011/4/e126/
doi: 10.2196/jmir.1923
PMID: 22209829

chigeorgetam@gmail.com Switch account
Not shared

* Indicates required question

Your name *
First Last

Cheuk Chi Tam

Primary Affiliation (short), City, Country *
University of Toronto, Toronto, Canada

University of South Carolina, Columbia, South (

Your e-mail address *
abc@gmail.com

ctam@mailbox.sc.edu

Title of your manuscript *
Provide the (draft) title of your manuscript.

Theory-based social media intervention for non-medical use of prescription opioids in young adults: Protocol for a randomized control trial

Name of your App/Software/Intervention *
If there is a short and a long/alternate name, write the short name first and add the long name in brackets.

E-NEW (Elevate Young Adults: Network for Em

Evaluated Version (if any)
e.g. "V1", "Release 2017-03-01", "Version 2.0.27913"

Your answer

Language(s) *
What language is the intervention/app in? If multiple languages are available, separate by comma (e.g. "English, French")

English

URL of your Intervention Website or App
e.g. a direct link to the mobile app on app in appstore (itunes, Google Play), or URL of the website. If the intervention is a DVD or hardware, you can also link to an Amazon page.

Your answer

URL of an image/screenshot (optional)

Your answer

Accessibility *
Can an enduser access the intervention presently?

- ( ) access is free and open
- (x) access only for special usergroups, not open
- ( ) access is open to everyone, but requires payment/subscription/in-app purchases
- ( ) app/intervention no longer accessible
- ( ) Other:

Primary Medical Indication/Disease/Condition *
e.g. "Stress", "Diabetes", or define the target group in brackets after the condition, e.g. "Autism (Parents of children with)", "Alzheimers (Informal Caregivers of)"

"young adults", "engaged in non-medical use o

Primary Outcomes measured in trial *
comma-separated list of primary outcomes reported in the trial

Intervention feasibility, acceptability, and usab

Secondary/other outcomes
Are there any other outcomes the intervention is expected to affect?

Non-medical use of prescription opioids (NMUPO), NMUPO knowledge, outcome expectancies, NMUPO risk perception, self-efficacy, psychological distress, resilience, and other substance use

Recommended "Dose" *
What do the instructions for users say on how often the app should be used?

- ( ) Approximately Daily
- (x) Approximately Weekly
- ( ) Approximately Monthly
- ( ) Approximately Yearly
- ( ) "as needed"
- ( ) Other:

Approx. Percentage of Users (starters) still using the app as recommended after 3 months *

- (x) unknown / not evaluated
- ( ) 0-10%
- ( ) 11-20%
- ( ) 21-30%
- ( ) 31-40%
- ( ) 41-50%
- ( ) 51-60%
- ( ) 61-70%
- ( ) 71%-80%
- ( ) 81-90%
- ( ) 91-100%
- ( ) Other:

Overall, was the app/intervention effective? *

- ( ) yes: all primary outcomes were significantly better in intervention group vs control
- ( ) partly: SOME primary outcomes were significantly better in intervention group vs control
- ( ) no statistically significant difference between control and intervention
- ( ) potentially harmful: control was significantly better than intervention in one or more outcomes
- ( ) inconclusive: more research is needed
- (x) Other: It is a proposed study. The intervention has not yet conducted.

Article Preparation Status/Stage *
At which stage in your article preparation are you currently (at the time you fill in this form)

- ( ) not submitted yet - in early draft status
- ( ) not submitted yet - in late draft status, just before submission
- ( ) submitted to a journal but not reviewed yet
- (x) submitted to a journal and after receiving initial reviewer comments
- ( ) submitted to a journal and accepted, but not published yet
- ( ) published
- ( ) Other:

Journal *
If you already know where you will submit this paper (or if it is already submitted), please provide the journal name (if it is not JMIR, provide the journal name under "other")

- ( ) not submitted yet / unclear where I will submit this
- ( ) Journal of Medical Internet Research (JMIR)
- ( ) JMIR mHealth and UHealth
- ( ) JMIR Serious Games
- ( ) JMIR Mental Health
- ( ) JMIR Public Health
- ( ) JMIR Formative Research
- ( ) Other JMIR sister journal
- (x) Other: JMIR Research Protocols

Is this a full powered effectiveness trial or a pilot/feasibility trial? *

- (x) Pilot/feasibility
- ( ) Fully powered

Manuscript tracking number *
If this is a JMIR submission, please provide the manuscript tracking number under "other" (The ms tracking number can be found in the submission acknowledgement email, or when you login as author in JMIR. If the paper is already published in JMIR, then the ms tracking number is the four-digit number at the end of the DOI, to be found at the bottom of each published article in JMIR)

- ( ) no ms number (yet) / not (yet) submitted to / published in JMIR
- (x) Other: 65847

TITLE AND ABSTRACT

1a) TITLE: Identification as a randomized trial in the title

1a) Does your paper address CONSORT item 1a? *
I.e does the title contain the phrase "Randomized Controlled Trial"? (if not, explain the reason under "other")

- (x) yes
- ( ) Other:

1a-i) Identify the mode of delivery in the title
Identify the mode of delivery. Preferably use "web-based" and/or "mobile" and/or "electronic game" in the title. Avoid ambiguous terms like "online", "virtual", "interactive". Use "Internet-based" only if Intervention includes non-web-based Internet components (e.g. email), use "computer-based" or "electronic" only if offline products are used. Use "virtual" only in the context of "virtual reality" (3-D worlds). Use "online" only in the context of "online support groups". Complement or substitute product names with broader terms for the class of products (such as "mobile" or "smart phone" instead of "iphone"), especially if the application runs on different platforms.

subitem not at all important 1 ( ) 2 ( ) 3 ( ) 4 ( ) 5 ( ) essential

Does your paper address subitem 1a-i? *
Copy and paste relevant sections from manuscript title (include quotes in quotation marks "like this" to indicate direct quotes from your manuscript), or elaborate on this item by providing additional information not in the ms, or briefly explain why the item is not applicable/relevant for your study

"Social media intervention"

1a-ii) Non-web-based components or important co-interventions in title
Mention non-web-based components or important co-interventions in title, if any (e.g., "with telephone support").

subitem not at all important 1 ( ) 2 ( ) 3 ( ) 4 ( ) 5 ( ) essential

Does your paper address subitem 1a-ii?
Copy and paste relevant sections from manuscript title (include quotes in quotation marks "like this" to indicate direct quotes from your manuscript), or elaborate on this item by providing additional information not in the ms, or briefly explain why the item is not applicable/relevant for your study

Your answer

1a-iii) Primary condition or target group in the title
Mention primary condition or target group in the title, if any (e.g., "for children with Type I Diabetes") Example: A Web-based and Mobile Intervention with Telephone Support for Children with Type I Diabetes: Randomized Controlled Trial

subitem not at all important 1 ( ) 2 ( ) 3 ( ) 4 ( ) 5 ( ) essential

Does your paper address subitem 1a-iii? *
Copy and paste relevant sections from manuscript title (include quotes in quotation marks "like this" to indicate direct quotes from your manuscript), or elaborate on this item by providing additional information not in the ms, or briefly explain why the item is not applicable/relevant for your study

"Young adults": We do not specify conditions since the intervention targets young adults engaged in non-medical use of prescription opioids but has no diagnoses of substance use disorders.

1b) ABSTRACT: Structured summary of trial design, methods, results, and conclusions
NPT extension: Description of experimental treatment, comparator, care providers, centers, and blinding status.

1b-i) Key features/functionalities/components of the intervention and comparator in the METHODS section of the ABSTRACT
Mention key features/functionalities/components of the intervention and comparator in the abstract. If possible, also mention theories and principles used for designing the site. Keep in mind the needs of systematic reviewers and indexers by including important synonyms. (Note: Only report in the abstract what the main paper is reporting. If this information is missing from the main body of text, consider adding it)

subitem not at all important 1 ( ) 2 ( ) 3 ( ) 4 ( ) 5 ( ) essential

Does your paper address subitem 1b-i? *
Copy and paste relevant sections from the manuscript abstract (include quotes in quotation marks "like this" to indicate direct quotes from your manuscript), or elaborate on this item by providing additional information not in the ms, or briefly explain why the item is not applicable/relevant for your study

"Qualitative findings will be used for developing a peer-led social media intervention to reduce NMUPO among young adults by integrating promising psychotherapy principles and incorporating with well-trained recovery coaches."

1b-ii) Level of human involvement in the METHODS section of the ABSTRACT
Clarify the level of human involvement in the abstract, e.g., use phrases like "fully automated" vs. "therapist/nurse/care provider/physician-assisted" (mention number and expertise of providers involved, if any). (Note: Only report in the abstract what the main paper is reporting. If this information is missing from the main body of text, consider adding it)

subitem not at all important 1 ( ) 2 ( ) 3 ( ) 4 ( ) 5 ( ) essential

Does your paper address subitem 1b-ii?
Copy and paste relevant sections from the manuscript abstract (include quotes in quotation marks "like this" to indicate direct quotes from your manuscript), or elaborate on this item by providing additional information not in the ms, or briefly explain why the item is not applicable/relevant for your study

"Developing a peer-led social media intervention to reduce NMUPO among young adults by integrating promising psychotherapy principles and incorporating with well-trained recovery coaches."

1b-iii) Open vs. closed, web-based (self-assessment) vs. face-to-face assessments in the METHODS section of the ABSTRACT
Mention how participants were recruited (online vs. offline), e.g., from an open access website or from a clinic or a closed online user group (closed usergroup trial), and clarify if this was a purely web-based trial, or there were face-to-face components (as part of the intervention or for assessment). Clearly say if outcomes were self-assessed through questionnaires (as common in web-based trials). Note: In traditional offline trials, an open trial (open-label trial) is a type of clinical trial in which both the researchers and participants know which treatment is being administered. To avoid confusion, use "blinded" or "unblinded" to indicated the level of blinding instead of "open", as "open" in web-based trials usually refers to "open access" (i.e. participants can self-enrol). (Note: Only report in the abstract what the main paper is reporting. If this information is missing from the main body of text, consider adding it)

subitem not at all important 1 ( ) 2 ( ) 3 ( ) 4 ( ) 5 ( ) essential

Does your paper address subitem 1b-iii?
Copy and paste relevant sections from the manuscript abstract (include quotes in quotation marks "like this" to indicate direct quotes from your manuscript), or elaborate on this item by providing additional information not in the ms, or briefly explain why the item is not applicable/relevant for your study

Your answer

1b-iv) RESULTS section in abstract must contain use data
Report number of participants enrolled/assessed in each group, the use/uptake of the intervention (e.g., attrition/adherence metrics, use over time, number of logins etc.), in addition to primary/secondary outcomes. (Note: Only report in the abstract what the main paper is reporting. If this information is missing from the main body of text, consider adding it)

subitem not at all important 1 ( ) 2 ( ) 3 ( ) 4 ( ) 5 ( ) essential

Does your paper address subitem 1b-iv?
Copy and paste relevant sections from the manuscript abstract (include quotes in quotation marks "like this" to indicate direct quotes from your manuscript), or elaborate on this item by providing additional information not in the ms, or briefly explain why the item is not applicable/relevant for your study

Your answer

1b-v) CONCLUSIONS/DISCUSSION in abstract for negative trials
Conclusions/Discussions in abstract for negative trials: Discuss the primary outcome - if the trial is negative (primary outcome not changed), and the intervention was not used, discuss whether negative results are attributable to lack of uptake and discuss reasons. (Note: Only report in the abstract what the main paper is reporting. If this information is missing from the main body of text, consider adding it)

subitem not at all important 1 ( ) 2 ( ) 3 ( ) 4 ( ) 5 ( ) essential

Does your paper address subitem 1b-v?
Copy and paste relevant sections from the manuscript abstract (include quotes in quotation marks "like this" to indicate direct quotes from your manuscript), or elaborate on this item by providing additional information not in the ms, or briefly explain why the item is not applicable/relevant for your study

Your answer

INTRODUCTION

2a) In INTRODUCTION: Scientific background and explanation of rationale

2a-i) Problem and the type of system/solution
Describe the problem and the type of system/solution that is object of the study: intended as stand-alone intervention vs. incorporated in broader health care program? Intended for a particular patient population? Goals of the intervention, e.g., being more cost-effective to other interventions, replace or complement other solutions? (Note: Details about the intervention are provided in "Methods" under 5)

subitem not at all important 1 ( ) 2 ( ) 3 ( ) 4 ( ) 5 ( ) essential

Does your paper address subitem 2a-i? *
Copy and paste relevant sections from the manuscript (include quotes in quotation marks "like this" to indicate direct quotes from your manuscript), or elaborate on this item by providing additional information not in the ms, or briefly explain why the item is not applicable/relevant for your study

"Aim 1
Conduct a formative study to inform a theory-based social media intervention for NMUPO among young adults in the US. By collaborating with 4 peer leaders trained as 'recovery coaches', the proposed study will recruit 30 US young adults (aged 18-25) who engage in NMUPO from social media (e.g., Instagram) and conduct in-depth semi-structured interviews to explore psychosocial contents associated with NMUPO. The interviews will be guided by the IMB model.
Aim 2
Develop a theory-based social intervention to reduce NMUPO among young adults. The intervention will be developed upon findings from the formative study and based on several promising strategies (e.g., peer support group, ambivalence resolving techniques, interactive training modules, virtual goal-setting, and video modeling). It will be delivered via Instagram private groups by peer leaders, with close supervision provided by experts and clinicians in the fields of health psychology and addiction medicine.
Aim 3
Test feasibility and preliminary efficacy of the theory-based social media intervention with a randomized controlled design for NMUPO among young adults. Participants who engage in recent NMUPO will be randomly assigned to one of two parallel groups, including the intervention group (n = 35) and control group (n = 35), using a 1:1 allocation ratio. The intervention will be evaluated in terms of feasibility, usability, acceptability, engagement, dose, and preliminary efficacy using mixed methods (e.g., surveys, EMA, Paradata, semi-structured interviews). During the intervention, EMA (a prompt every two days) will assess acceptability and usability of individual modules. Preliminary efficacy will be tested on behavioral outcomes (i.e., past-month NMUPO) and psychosocial factors (e.g., outcome expectancy, self-efficacy, action/coping planning, resilience, psychiatric symptoms). "

2a-ii) Scientific background, rationale: What is known about the (type of) system
Scientific background, rationale: What is known about the (type of) system that is the object of the study (be sure to discuss the use of similar systems for other conditions/diagnoses, if appropiate), motivation for the study, i.e. what are the reasons for and what is the context for this specific study, from which stakeholder viewpoint is the study performed, potential impact of findings [2]. Briefly justify the choice of the comparator.

subitem not at all important 1 ( ) 2 ( ) 3 ( ) 4 ( ) 5 ( ) essential

Does your paper address subitem 2a-ii? *
Copy and paste relevant sections from the manuscript (include quotes in quotation marks "like this" to indicate direct quotes from your manuscript), or elaborate on this item by providing additional information not in the ms, or briefly explain why the item is not applicable/relevant for your study

"Non-medical use of prescription opioids (NMUPO) is a critical public health concern in the United States (US), and young adults are particularly vulnerable to this behavior. NMUPO refers to aberrant use of prescription opioids in a manner other than as prescribed.[1-3] Substance use literature has paid increasing attention to NMUPO owing to its adverse consequences. National US data have shown a substantial increase of opioid-related overdose deaths from <5,000 in 1999 to >100,000 in 2022[4, 5]. NMUPO has also led to extensive medical costs in the US (78.5 billion annually)[6]. Young adults (aged 18-25) are at high-risk for NMUPO. In 2019, 5.5% of young adults in the US engaged in past-year NMUPO, compared to 2.3-3.5% for other age groups. This rate was even higher among those who were not enrolled in colleges (6.3%)[7]. Besides risk of overdose and dependence, NMUPO is associated with other negative outcomes, including health-jeopardizing behaviors (e.g., driving under the influence), sexual victimization, and suicide attempts [1, 8-11]. In response to this issue, many US jurisdictions have launched initiatives to monitor prescription opioids [12]. Despite an obvious reduction of prescribing rate (e.g., 80% in 2009 to 59% in 2018)[12], NMUPO-related deaths remain high (> 100,000 in 2022). Notably, NMUPO is strongly linked with initiation of heroin and synthetic opioid use (e.g., fentanyl) in young adults, posing a substantial risk for development of substance use disorder (SUD) and overdose [13]. Hence, interventions targeting young adults are urgently needed to address NMUPO, and they should be delivered beyond the college population [14].
Interventions for NMUPO in young adults should take psychosocial factors into account. NMUPO literature has identified several important psychosocial factors associated with NMUPO in young adults, including perceived stress (e.g., academic strain, traumatic stress), psychiatric distress (e.g., depression, anxiety), perception of risk, peer and family influences, and sensation seeking [15-18]. My previous research on NMUPO in college students has indicated several additional factors that reduce risk for NMUPO, such as psychological resilience, positive coping, outcome expectancies, and self-esteem [10, 19-21]. Importantly, psychosocial factors robustly contribute to NMUPO. A meta-analysis study revealed that pooled effect sizes of psychosocial factors on NMUPO (ORs = 2.14 to 2.45) were higher than those of somatic symptoms (ORs = 0.81 to 1.76), which are traditionally viewed as major NMUPO determinants [22]. These findings indicate that interventions should be guided by a structured framework between psychosocial factors and health actions for NMUPO.
There are a handful of interventions that have been recently developed to address psychosocial aspects of NMUPO in young populations [23-25]. Although these programs have shown some efficacy, they encounter numerous challenges. First, most programs have been adapted from existing substance use interventions that are not originally designed for NMUPO. For example, some contents specific to opioids (e.g., the risk for overdose and pain relief expectancy) are absent from these programs, yet these factors significantly contribute to health action related to NMUPO. Second, most programs were delivered in schools and failed to target young adults who did not attend college or who were difficult-to-reach. Third, the flexibility of these interventions was limited since they were delivered by professionals in an in-person setting. Further, literature on substance misuse prevention in young adults has revealed several drawbacks to the in-person context, such as limited trust and fear of being stigmatized [26]. These challenges highlight the critical need for an innovative intervention that is based on psychosocial theory, tailored to the NMUPO context, and flexible in real world settings."

2b) In INTRODUCTION: Specific objectives or hypotheses

Does your paper address CONSORT subitem 2b? *
Copy and paste relevant sections from the manuscript (include quotes in quotation marks "like this" to indicate direct quotes from your manuscript), or elaborate on this item by providing additional information not in the ms, or briefly explain why the item is not

applicable/relevant for your study

"Aim 1
Conduct a formative study to inform a theory-based social media intervention for NMUPO among young adults in the US. By collaborating with 4 peer leaders trained as 'recovery coaches', the proposed study will recruit 30 US young adults (aged 18-25) who engage in NMUPO from social media (e.g., Instagram) and conduct in-depth semi-structured interviews to explore psychosocial contents associated with NMUPO. The interviews will be guided by the IMB model.
Aim 2
Develop a theory-based social intervention to reduce NMUPO among young adults. The intervention will be developed upon findings from the formative study and based on several promising strategies (e.g., peer support group, ambivalence resolving techniques, interactive training modules, virtual goal-setting, and video modeling). It will be delivered via Instagram private groups by peer leaders, with close supervision provided by experts and clinicians in the fields of health psychology and addiction medicine.
Aim 3
Test feasibility and preliminary efficacy of the theory-based social media intervention with a randomized controlled design for NMUPO among young adults. Participants who engage in recent NMUPO will be randomly assigned to one of two parallel groups, including the intervention group (n = 35) and control group (n = 35), using a 1:1 allocation ratio. The intervention will be evaluated in terms of feasibility, usability, acceptability, engagement, dose, and preliminary efficacy using mixed methods (e.g., surveys, EMA, Paradata, semi-structured interviews). During the intervention, EMA (a prompt every two days) will assess acceptability and usability of individual modules. Preliminary efficacy will be tested on behavioral outcomes (i.e., past-month NMUPO) and psychosocial factors (e.g., outcome expectancy, self-efficacy, action/coping planning, resilience, psychiatric symptoms). "

METHODS

3a) Description of trial design (such as parallel, factorial) including allocation ratio

Does your paper address CONSORT subitem 3a? *

Copy and paste relevant sections from the manuscript (include quotes in quotation marks "like this" to indicate direct quotes from your manuscript), or elaborate on this item by providing additional information not in the ms, or briefly explain why the item is not applicable/relevant for your study

"Participants will be randomly assigned to the parallel intervention (n = 35) or control group (n = 35) using computer-generated random numbers. Participants are blinded to the randomized assignments. "

3b) Important changes to methods after trial commencement (such as eligibility criteria), with reasons

Does your paper address CONSORT subitem 3b? *

Copy and paste relevant sections from the manuscript (include quotes in quotation marks "like this" to indicate direct quotes from your manuscript), or elaborate on this item by providing additional information not in the ms, or briefly explain why the item is not applicable/relevant for your study

No changes were made because of eligibility criteria.

3b-i) Bug fixes, Downtimes, Content Changes

Bug fixes, Downtimes, Content Changes: ehealth systems are often dynamic systems. A description of changes to methods therefore also includes important changes made on the intervention or comparator during the trial (e.g., major bug fixes or changes in the functionality or content) (5-iii) and other "unexpected events" that may have influenced study design such as staff changes, system failures/downtimes, etc. [2].

	1	2	3	4	5	
subitem not at all important	○	○	○	○	○	essential

Does your paper address subitem 3b-i?

Copy and paste relevant sections from the manuscript (include quotes in quotation marks "like this" to indicate direct quotes from your manuscript), or elaborate on this item by providing additional information not in the ms, or briefly explain why the item is not applicable/relevant for your study

Your answer

4a) Eligibility criteria for participants

Does your paper address CONSORT subitem 4a? *

Copy and paste relevant sections from the manuscript (include quotes in quotation marks "like this" to indicate direct quotes from your manuscript), or elaborate on this item by providing additional information not in the ms, or briefly explain why the item is not applicable/relevant for your study

"Participants for the formative study and intervention trial will be those who meet the inclusion criteria: (1) aged 18-25; (2) US residents; (3) engaged in NMUPO in the past three months; (4) using Instagram at least 3 times a week = in the past three months. NMUPO refers to the occurrence of following behaviors (one time or more): (1) taking a prescription opioid without a prescription; (2) taking more doses than as prescribed; (3) using for a non-medical reason (e.g., getting high) [1-3]. Individuals will be excluded if they report: (1) receiving substance use interventions in the past three months, (2) are diagnosed with SUD; or (3) are not proficient in English."

4a-i) Computer / Internet literacy

Computer / Internet literacy is often an implicit "de facto" eligibility criterion - this should be explicitly clarified.

	1	2	3	4	5	
subitem not at all important	○	○	○	○	○	essential

Does your paper address subitem 4a-i?

Copy and paste relevant sections from the manuscript (include quotes in quotation marks "like this" to indicate direct quotes from your manuscript), or elaborate on this item by providing additional information not in the ms, or briefly explain why the item is not applicable/relevant for your study

Your answer

4a-ii) Open vs. closed, web-based vs. face-to-face assessments:

Open vs. closed, web-based vs. face-to-face assessments: Mention how participants were recruited (online vs. offline), e.g., from an open access website or from a clinic, and clarify if this was a purely web-based trial, or there were face-to-face components (as part of the intervention or for assessment), i.e., to what degree got the study team to know the participant. In online-only trials, clarify if participants were quasi-anonymous and whether having multiple identities was possible or whether technical or logistical measures (e.g., cookies, email confirmation, phone calls) were used to detect/prevent these.

	1	2	3	4	5	
subitem not at all important	○	○	○	○	○	essential

Does your paper address subitem 4a-ii? *

Copy and paste relevant sections from the manuscript (include quotes in quotation marks "like this" to indicate direct quotes from your manuscript), or elaborate on this item by providing additional information not in the ms, or briefly explain why the item is not applicable/relevant for your study

"The entire recruitment procedure is presented in Figure 2. Recruitment will be conducted using two strategies: (1) peer outreach and (2) advertising campaign. For the peer outreach approach, peer leaders will distribute recruitment advertisements (ads) via online social networking (e.g., posting Ads on their social media accounts, inviting subscribers to re-post the Ads). Peers can also send direct invitations to young adults who they personally know to be eligible for the study. For advertising campaign approaches, we will target the accounts (on Instagram/Facebook) of colleges, local young adult communities (e.g., trade unions), and substance use support groups. Upon approval from their group administrators, we will post ads in the groups. Also, we will develop targeted advertising using Ads Manager [57]. The ads will display research-relevant images, hashtags, and headings, which are tailored to engaging contents for young adults. To ensure the appropriateness, the ads will be reviewed by a Community Advisory Board. The ads for Aim 3 will be improved in line with formative research. The Ads Manager will also determine the range of the ad campaign in terms of age, time, and locations (i.e., US). The designed ads will be delivered in various formats dependent on the platforms, such as linear posts within personal feeds on Instagram, News Feeds on Facebook, and Stories on Snapchat. To ensure representativeness, recruitment will be stratified based on key demographics (e.g., gender, race/ethnicity, and education) in line with the latest US national data on NMUPO [58]. The Ads will navigate potential participants to an online sign-up survey on RedCap, which is a secured online platform monitored by USC. "
"The web-based pre- and post-intervention surveys will be developed and administered on RedCap [94] to measure demographic variables, as well as psychosocial and behavioral factors outlined in the IMB-based conceptual framework. Factors include NMUPO behaviors (past-3-month NMUPO), and information- and motivation-related factors (i.e., NMUPO knowledge, NMUPO risk perception, outcome expectancies, self-efficacy, psychological distress, resilience, and other substance use). The web-based surveys will be conducted among participants in both the intervention and control groups. Participants will receive survey links via inbox messages to the Instagram accounts that they provide for the study. The pre-intervention survey will be sent two weeks before the intervention (baseline), and the post-intervention survey will be sent in the week immediately following the intervention (12-week follow-up). The survey will be self-administered and take approximately 20-30 minutes to complete. If participants cannot complete the surveys at one time, they can save their responses, and the platform will generate a code that allows them to resume the survey within the same week. The survey will also provide forward and back buttons, allowing participants to review and change their responses before they submit their surveys. Study IDs and filling time will be collected for determining duplicate entries. E-gift-cards will be provided for the completion of surveys (baseline and 12 weeks; $30 each)."

4a-iii) Information giving during recruitment

Information given during recruitment. Specify how participants were briefed for recruitment and in the informed consent procedures (e.g., publish the informed consent documentation as appendix, see also item X26), as this information may have an effect on user self-selection, user expectation and may also bias results.

	1	2	3	4	5	
subitem not at all important	○	○	○	○	○	essential

Does your paper address subitem 4a-iii?

Copy and paste relevant sections from the manuscript (include quotes in quotation marks "like this" to indicate direct quotes from your manuscript), or elaborate on this item by providing additional information not in the ms, or briefly explain why the item is not applicable/relevant for your study

Your answer

4b) Settings and locations where the data were collected

Does your paper address CONSORT subitem 4b? *

Copy and paste relevant sections from the manuscript (include quotes in quotation marks "like this" to indicate direct quotes from your manuscript), or elaborate on this item by providing additional information not in the ms, or briefly explain why the item is not applicable/relevant for your study

"Aim 1: Formative study
Young adults who are social media users (e.g., Instagram and Facebook) and indicate NMUPO will be invited to online 60-minute individual semi-structured in-depth interviews. The interview guide will be based on the IMB model and will aim to: (1) identify NMUPO patterns (e.g., drug classes, individual behaviors); (2) understand psychosocial factors contributing to reduce NMUPO; (2) extract vivid examples related to psychosocial content; (3) identify planning processes and coping skills facilitating the change; (4) assess the feasibility/acceptability of social media to manage NMUPO; (5) review recruitment ads. This will provide the foundational knowledge and materials to develop specific content (e.g., video and posts) for the intervention. The interviewers will be trained research assistants from the fields of psychology and/or public health. As suggested by the guideline of qualitative research [62], the sample size will be 30 young adults. Participants will receive $50 e-gift cards as study incentives."

"Web-based pre- and post-intervention surveys
The web-based pre- and post-intervention surveys will be developed and administered on RedCap [94] to measure demographic variables, as well as psychosocial and behavioral factors outlined in the IMB-based conceptual framework. Factors include NMUPO behaviors (past-3-month NMUPO), and information- and motivation-related factors (i.e., NMUPO knowledge, NMUPO risk perception, outcome expectancies, self-efficacy, psychological distress, resilience, and other substance use). The web-based surveys will be conducted among participants in both the intervention and control groups. Participants will receive survey links via inbox messages to the Instagram accounts that they provide for the study. The pre-intervention survey will be sent two weeks before the intervention (baseline), and the post-intervention survey will be sent in the week immediately following the intervention (12-week follow-up). The survey will be self-administered and take approximately 20-30 minutes to complete. If participants cannot complete the surveys at one time, they can save their responses, and the platform will generate a code that allows them to resume the survey within the same week. The survey will also provide forward and back buttons, allowing participants to review and change their responses before they submit their surveys. Study IDs and filling time will be collected for determining duplicate entries. E-gift-cards will be provided for the completion of surveys (baseline and 12 weeks; $30 each).
Ecological momentary assessment (EMA)
EMA will be employed through brief evening surveys to screen the prompt acceptability and usability (USE questionnaire)[95] , and dose of particular modules in the intervention group. The EMA survey will be conducted once in every two days during the intervention. The daily EMA is widely used in behavioral trials [39]. Data will be collected through 20-second survey on a smartphone application (ExpiWell), which is user-friendly and customizable for EMA development and implementation. [96]A prompt will be sent at the moment for assessment and tailored for the intervention materials in the past two days. A reminder will be provided if no response is made in 30 minutes. For safety purposes, if an EMA occurs during an incompatible activity (e.g., driving), participants will be instructed to ignore/postpone the prompt. A prorated incentive (e-gift card, $25 total) will be offered depending on a participant's EMA completion.
Incentives
Post-intervention semi-structured interviews
At week 12, participants will join semi-structural interviews measuring: (1) feasibility, usability, and acceptability; (2) reactions to the contents, format, concepts, visual presentation, assessments (survey and EMA), and adaptation to other platforms. The interview guide will be based on mHealth studies [97]. The interview will be audio-recorded and take approximately 60 minutes through an online meeting application (e.g., Zoom). Upon completion of the interview, participants will receive $30 e-gift cards as incentives."

4b-i) Report if outcomes were (self-)assessed through online questionnaires

Clearly report if outcomes were (self-)assessed through online questionnaires (as common in web-based trials) or otherwise.

	1	2	3	4	5	
subitem not at all important	○	○	○	○	○	essential

Does your paper address subitem 4b-i? *

Copy and paste relevant sections from the manuscript (include quotes in quotation marks "like this" to indicate direct quotes from your manuscript), or elaborate on this item by providing additional information not in the ms, or briefly explain why the item is not applicable/relevant for your study

"The survey will be self-administered and take approximately 20-30 minutes to complete. If participants cannot complete the surveys at one time, they can save their responses, and the platform will generate a code that allows them to resume the survey within the same week. The survey will also provide forward and back buttons, allowing participants to review and change their responses before they submit their surveys. Study IDs and filling time will be collected for determining duplicate entries. E-gift-cards will be provided for the completion of surveys (baseline and 12 weeks; $30 each)."

4b-ii) Report how institutional affiliations are displayed

Report how institutional affiliations are displayed to potential participants [on ehealth media], as affiliations with prestigious hospitals or universities may affect volunteer rates, use, and reactions with regards to an intervention.(Not a required item – describe only if this may bias results)

	1	2	3	4	5	
subitem not at all important	○	○	○	○	○	essential

Does your paper address subitem 4b-ii?

Copy and paste relevant sections from the manuscript (include quotes in quotation marks "like this" to indicate direct quotes from your manuscript), or elaborate on this item by providing additional information not in the ms, or briefly explain why the item is not applicable/relevant for your study

Your answer

5) The interventions for each group with sufficient details to allow replication, including how and when they were actually administered

5-i) Mention names, credential, affiliations of the developers, sponsors, and owners

Mention names, credential, affiliations of the developers, sponsors, and owners [6] (if authors/evaluators are owners or developer of the software, this needs to be declared in a "Conflict of interest" section or mentioned elsewhere in the manuscript).

	1	2	3	4	5	
subitem not at all important	○	○	○	○	○	essential

Does your paper address subitem 5-i?

Copy and paste relevant sections from the manuscript (include quotes in quotation marks "like this" to indicate direct quotes from your manuscript), or elaborate on this item by providing additional information not in the ms, or briefly explain why the item is not applicable/relevant for your study

Your answer

5-ii) Describe the history/development process

Describe the history/development process of the application and previous formative evaluations (e.g., focus groups, usability testing), as these will have an impact on adoption/use rates and help with interpreting results.

	1	2	3	4	5	
subitem not at all important	○	○	○	○	○	essential

Does your paper address subitem 5-ii?

Copy and paste relevant sections from the manuscript (include quotes in quotation marks "like this" to indicate direct quotes from your manuscript), or elaborate on this item by providing additional information not in the ms, or briefly explain why the item is not applicable/relevant for your study

Your answer

5-iii) Revisions and updating

Revisions and updating. Clearly mention the date and/or version number of the application/intervention (and comparator, if applicable) evaluated, or describe whether the intervention underwent major changes during the evaluation process, or whether the development and/or content was "frozen" during the trial. Describe dynamic components such as news feeds or changing content which may have an impact on the replicability of the intervention (for unexpected events see item 3b).

	1	2	3	4	5	
subitem not at all important	○	○	○	○	○	essential

Does your paper address subitem 5-iii?

Copy and paste relevant sections from the manuscript (include quotes in quotation marks "like this" to indicate direct quotes from your manuscript), or elaborate on this item by providing additional information not in the ms, or briefly explain why the item is not applicable/relevant for your study

Your answer

5-iv) Quality assurance methods

Provide information on quality assurance methods to ensure accuracy and quality of information provided [1], if applicable.

	1	2	3	4	5	
subitem not at all important	○	○	○	○	○	essential

Does your paper address subitem 5-iv?

Copy and paste relevant sections from the manuscript (include quotes in quotation marks "like this" to indicate direct quotes from your manuscript), or elaborate on this item by providing additional information not in the ms, or briefly explain why the item is not applicable/relevant for your study

Your answer

5-v) Ensure replicability by publishing the source code, and/or providing screenshots/screen-capture video, and/or providing flowcharts of the algorithms used

Ensure replicability by publishing the source code, and/or providing screenshots/screen-capture video, and/or providing flowcharts of the algorithms used. Replicability (i.e., other researchers should in principle be able to replicate the study) is a hallmark of scientific reporting.

	1	2	3	4	5	
subitem not at all important	○	○	○	○	○	essential

Does your paper address subitem 5-v?

Copy and paste relevant sections from the manuscript (include quotes in quotation marks "like this" to indicate direct quotes from your manuscript), or elaborate on this item by providing additional information not in the ms, or briefly explain why the item is not applicable/relevant for your study

Your answer

5-vi) Digital preservation

Digital preservation: Provide the URL of the application, but as the intervention is likely to change or disappear over the course of the years; also make sure the intervention is archived (Internet Archive, webcitation.org, and/or publishing the source code or screenshots/videos alongside the article). As pages behind login screens cannot be archived, consider creating demo pages which are accessible without login.

	1	2	3	4	5	
subitem not at all important	○	○	○	○	○	essential

Does your paper address subitem 5-vi?

Copy and paste relevant sections from the manuscript (include quotes in quotation marks "like this" to indicate direct quotes from your manuscript), or elaborate on this item by providing additional information not in the ms, or briefly explain why the item is not applicable/relevant for your study

Your answer

5-vii) Access

Access: Describe how participants accessed the application, in what setting/context, if they had to pay (or were paid) or not, whether they had to be a member of specific group. If known, describe how participants obtained "access to the platform and Internet" [1]. To ensure access for editors/reviewers/readers, consider to provide a "backdoor" login account or demo mode for reviewers/readers to explore the application (also important for archiving purposes, see vi).

	1	2	3	4	5	
subitem not at all important	○	○	○	○	○	essential

Does your paper address subitem 5-vii? *

Copy and paste relevant sections from the manuscript (include quotes in quotation marks "like this" to indicate direct quotes from your manuscript), or elaborate on this item by providing additional information not in the ms, or briefly explain why the item is not applicable/relevant for your study

"Intervention group will be guided to join Instagram peer-led private groups (n = 10-15 each). Participants will be asked to provide their Instagram accounts to their assigned peer leaders for participating in intervention activities. Peer leaders will set up private and independent groups through their Instagram accounts and will send direct invitations to their assigned to join groups. Once group membership is confirmed, peer leaders will post intervention materials and lead group activities according to the plan as developed in Aim 2. Private groups will synchronously deliver intervention packages over 12 weeks"

5-viii) Mode of delivery, features/functionalities/components of the intervention and comparator, and the theoretical framework

Describe mode of delivery, features/functionalities/components of the intervention and comparator, and the theoretical framework [6] used to design them (instructional strategy [1], behaviour change techniques, persuasive features, etc., see e.g., [7, 8] for terminology). This includes an in-depth description of the content (including where it is coming from and who developed it) [1]," whether [and how] it is tailored to individual circumstances and allows users to track their progress and receive feedback" [6]. This also includes a description of communication delivery channels and – if computer-mediated communication is a component – whether communication was synchronous or asynchronous [6]. It also includes information on presentation strategies [1], including page design principles, average amount of text on pages, presence of hyperlinks to other resources, etc. [1].

	1	2	3	4	5	
subitem not at all important	○	○	○	○	○	essential

Does your paper address subitem 5-viii? *

Copy and paste relevant sections from the manuscript (include quotes in quotation marks "like this" to indicate direct quotes from your manuscript), or elaborate on this item by providing additional information not in the ms, or briefly explain why the item is not applicable/relevant for your study

"To guide an intervention addressing psychosocial aspects of NMUPO, a theoretical framework (see Figure 1) was developed based on the information-motivation-behavioral skills (IMB) model [27]. The IMB model has been applauded for its strength in considering a straightforward path diagram for health behavior changes [28]. This model emphasizes that health behavior changes are driven by three arrays of psychosocial constructs—information, motivation, and behavioral skills. Information refers to knowledge contributing to a prerequisite for enacting the changes (e.g., misinformation of opioids). Motivation includes two belief components: personal motivation (e.g., outcome expectancies and risk perceptions) and social motivation (e.g., social norms and support). Behavioral skills stand for cognitive capacities (e.g., self-efficacy and resilience) and behavioral skill sets necessary for adopting the changes (e.g., coping/self-regulation skills) or for managing barriers (e.g., stress management)[27, 28]. Information and motivation can directly affect the target behavior, as well as indirectly affect it through acquisition of adjacent behavioral skills. Such a well-structured framework offers a clear guidance for intervention practices and suggests that intervention delivery could benefit from targeting information and motivation factors in the initial sessions, along with enhancing the role of behavioral skill training gradually in the intervention. For instance, initial efforts ought to address misinformation and outcome expectancies of substance use, and, as the intervention progresses, sessions should facilitate action by helping participants develop planning strategies and coping skills. Indeed, existing evidence has shown efficacy of IMB-guided interventions on reducing substance use (i.e., tobacco and illicit drugs) [29-32]. Thus, the IMB model is an ideal theoretical choice for developing an intervention for NMUPO."

"The proposed intervention components are guided by the IMB-based conceptual framework (see Figure 1), with each component targeting specific factors derived from three psychosocial constructs (i.e., information, motivation, and behavioral skills). As outlined in the framework, the pacing of intervention is structured to prioritize addressing information and motivation factors during the initial phases, while later sessions progressively promote behavioral skills training that contribute to psychological distress management and NMUPO reduction. Incorporating Instagram functions, the intervention platform is proposed to consist of four intervention modules, including: (1) NMUPO knowledge module; (2) self-care module; (3) virtual goal setting/monitoring module; (4) peer support module (see Figure 3). These are planned, and final contents will be informed by or modified in line with the formative research. The research team will work closely with the community advisory board and peer leaders to ensure appropriate contents and language. The intervention will last 12 weeks according to previous social media trials (typically 8-12 weeks) [37, 44, 79-86]. Clinicians will review the intervention materials and ensure the scope is appropriate. Contents will be prompted daily as suggested by the IMB model (e.g., initially focus on information and motivation factors and gradually enhance the role of behavioral skills). "

5-ix) Describe use parameters

Describe use parameters (e.g., intended "doses" and optimal timing for use). Clarify what instructions or recommendations were given to the user, e.g., regarding timing, frequency, heaviness of use, if any, or was the intervention used ad libitum.

	1	2	3	4	5	
subitem not at all important	○	○	○	○	○	essential

Does your paper address subitem 5-ix?

Copy and paste relevant sections from the manuscript (include quotes in quotation marks "like this" to indicate direct quotes from your manuscript), or elaborate on this item by providing additional information not in the ms, or briefly explain why the item is not applicable/relevant for your study

Your answer

5-x) Clarify the level of human involvement

Clarify the level of human involvement (care providers or health professionals, also technical assistance) in the e-intervention or as co-intervention (detail number and expertise of professionals involved, if any, as well as "type of assistance offered, the timing and frequency of the support, how it is initiated, and the medium by which the assistance is delivered". It may be necessary to distinguish between the level of human involvement required for the trial, and the level of human involvement required for a routine application outside of a RCT setting (discuss under item 21 – generalizability).

	1	2	3	4	5	
subitem not at all important	○	○	○	○	○	essential

Does your paper address subitem 5-x?

Copy and paste relevant sections from the manuscript (include quotes in quotation marks "like this" to indicate direct quotes from your manuscript), or elaborate on this item by providing additional information not in the ms, or briefly explain why the item is not applicable/relevant for your study

Your answer

5-xi) Report any prompts/reminders used

Report any prompts/reminders used: Clarify if there were prompts (letters, emails, phone calls, SMS) to use the application, what triggered them, frequency etc. It may be necessary to distinguish between the level of prompts/reminders required for the trial, and the level of prompts/reminders for a routine application outside of a RCT setting (discuss under item 21 – generalizability).

	1	2	3	4	5	
subitem not at all important	○	○	○	○	○	essential

Does your paper address subitem 5-xi? *

Copy and paste relevant sections from the manuscript (include quotes in quotation marks "like this" to indicate direct quotes from your manuscript), or elaborate on this item by providing additional information not in the ms, or briefly explain why the item is not applicable/relevant for your study

"Several strategies that are shown useful for enhancing engagement will be utilized: [82, 84, 89-92](1) reminder notifications (via Instagram/emails); (2) prompt replies to participants' messages; (3) biweekly prize draws of $25 gift cards for those who post, attend meetings, and reply on the panels. The intervention will be overseen by PI and a licensed clinical psychologist at the SSUDS-SC Center, under supervision from experts in addiction medicine and psychotherapy for SUD. They will provide preintervention training (e.g., skills in MI/CBT techniques and findings from Aim 1) and weekly guidance to peer leaders. They will also advise on referrals to link participants to licensed mental and behavioral health professionals in their states of residence if necessary. The doctoral-level psychology trainees at SSUDS-SC Center will support intervention delivery by: (1) ensuring conversations intervention-related and ban inappropriate contents; (2) promptly replying to participants' comments; and (3) monitoring participants' safety (e.g., emotional reactions to the posts and in the online group meetings) and offering referrals for outside psychological services, if needed. They will also be responsible for reporting adverse events to the PI if observed."

5-xii) Describe any co-interventions (incl. training/support)

Describe any co-interventions (incl. training/support): Clearly state any interventions that are provided in addition to the targeted eHealth intervention, as ehealth intervention may not be designed as stand-alone intervention. This includes training sessions and support

[1]. It may be necessary to distinguish between the level of training required for the trial, and the level of training for a routine application outside of a RCT setting (discuss under item 21 – generalizability.

	1	2	3	4	5	
subitem not at all important	○	○	○	○	○	essential

Does your paper address subitem 5-xii? *

Copy and paste relevant sections from the manuscript (include quotes in quotation marks "like this" to indicate direct quotes from your manuscript), or elaborate on this item by providing additional information not in the ms, or briefly explain why the item is not applicable/relevant for your study

N/A: This is a feasibility study of a new intervention which is not dependent on other exisiting and on-going interventions.

6a) Completely defined pre-specified primary and secondary outcome measures, including how and when they were assessed

Does your paper address CONSORT subitem 6a? *

Copy and paste relevant sections from the manuscript (include quotes in quotation marks "like this" to indicate direct quotes from your manuscript), or elaborate on this item by providing additional information not in the ms, or briefly explain why the item is not applicable/relevant for your study

"Measures
Mixed-method measures for primary outcomes: intervention feasibility outcomes
Multiple forms of data will be collected within 12 weeks during the intervention for the intervention feasibility evaluation.
Feasibility, acceptability, and usability
Mixed methods will be used. Acceptability will be assessed using EMA on Likert items that rate how helpful the module is (1 = not at all to 5 = extremely). Usability will be measured using EMA on the Usefulness, Satisfaction, and Ease of use (USE) questionnaire [95] which assesses perceived usefulness, satisfaction, and ease of use for the module. At week 12, participants will join semi-structural interviews measuring: (1) feasibility, usability, and acceptability; (2) reactions to the contents, format, concepts, visual presentation, assessments (survey and EMA), and adaptation to other platforms. The interview guide will be based on mHealth studies [97].
Engagement
Paradata on participants' interactions with specific modules will be collected, including their frequency of reactions (i.e., comments, 'likes', questions, and replies on specific posts) and personal posts to Instagram groups [37]. Retention will be assessed by calculating the percentage of participants engaging in the group at varying time points. Data will be collected in real time.
Dose of intervention
Participants' engagement in particular intervention modules will be measured in EMA surveys as guided by dose operationalization for mHealth interventions [98]. Dose for specific modules will be assessed in 3 domains (1 yes/no item each): (1) intervention actions (if viewing a post); (2) participant actions (if practicing a skill/completing an assignment); (3) behavioral target actions (if adopting skills outside of the intervention).
Web-based surveys for demographic variables and secondary outcomes: psychosocial and behavioral outcomes
The web-based surveys will measure demographic factors and psychosocial and behavioral factors outlined in the IMB-based conceptual model (Figure 2).
Demographic characteristics
In the baseline pre-intervention survey, participants will be asked to report their background information regarding gender (i.e., male, female, transgender, or other), race/ethnicity (e.g., Caucasian/White, African American/Black), age (years), socio-economic status, education, family/household characteristics, and physical health status (Patient Health Questionnaire-15) [99].
NMUPO
The past-three-month NMUPO will be measured using relevant items from the Tobacco, Alcohol, Prescription medication, and other Substance use (TAPS) tool [100]. The scale includes two sections, with a screener followed by a brief assessment. The screener contains one items asking frequency of NMUPO (i.e., used just for feeling, more than prescribed, or without a prescription) with five response options (daily or almost daily, weekly, monthly, less than monthly, or never). Participants with a response other than "never" will be led to the brief assessment including three dichotomous questions regarding their level of use, dependence, concern from others related to the past-three-month NMUPO. The sum score of the brief assessment will be calculated, with a higher score indicating a greater level of NMUPO.
NMUPO knowledge
Information about NMUPO and relevant topics, such as misconceptions, the role of fentanyl, naloxone, and Xyalazine, will be assessed using a the NMUPO knowledge scale [61]. This scale will be developed according to the measure in POP4Teens and the formative study. This scale is proposed to include 15 statements related to opioids or non-medical use of prescription opioids. Participant will be asked to determine if a statement is true or not (0 = False, 1 = True). Responses will then be rated by the research team, with higher scores indicating better knowledge of non-medical use of prescription opioids.
Outcome expectancies
Beliefs on positive or negative consequences of engaging in NMUPO will be assessed using a scale adapted from the Behaviors, Expectancies, Attitudes and College Health Questionnaire (BEACH-Q) [101]. A total of 50 Items will be scored on a five-point Likert-type scale ranging from 0 (Not at all) to 4 (Very often or always) with higher sum scores standing for the greater level of expectancies. The scale consists of eight dimensions related to specific expectancies of opioids, including pain reduction, tension reduction, academic preference, emotion enhancement, social enhancement, guilt and dependence, cognitive impairment, and physical discomfort.
NMUPO risk perception
Perceived vulnerability and severity of engaging in NMUPO will be assessed using the Perceived Risk Scale for Prescription Drug Abuse [102]. This scale includes 5 items with four rating options (1="strongly disagree" to 4 = "strongly agree"). The sum score will be generated, with higher score indicating a greater level of perception risk for NMUPO.
Action self-efficacy and coping self-efficacy
Perceived control on making actions to stop/reduce NMUPO or confidence on coping with barriers against the actions will be assessed using an adapted version of Self-Efficacy Scale [103]. A total of six Items will be rated on a 5-point Likert scale (1 = strongly disagree, 5 = strongly agree) and can be organized into 2 subscales (coping self-efficacy and action self-efficacy) with 3 items each. The sum scores will be generated for each subscale, with higher scores indicating greater levels of self-efficacy.
Psychological distress
Depression and anxiety symptoms in the past two weeks will be assessed using the Patient Health Questionnaire-9 (PHQ-9) [104]and General Anxiety Disorder-7 (GAD-7)[105], respectively. PHQ-9 is composed of nine items asking the frequency of depressive mood experiences (e.g., 'feeling down, depressed, and hopeless'), while GAD-7 comprises seven items assessing the frequency of feelings of nervousness or worry (e.g., 'Not being able to stop or control worrying'). Two scales have 4 response options ranging from 0 (not at all) to 4 (nearly every day). Sum scores for two scales will be generated, with higher scores indicating greater levels of depression or anxiety.
Resilience
The Connor-Davidson Resilience Scale (CD-RISD) will be utilized for assessing psychological resilience [106]. This scale has 25 items asking about personal capacities in response to stress, including tenacity, tolerance of negative affect, positive acceptance of change, and positive view of adversities. Items will be rated on a 5-point scale (1 = not at all to 5 = nearly all the time). The sum score will be generated, with a higher score indicating a greater level of resilience.
Other substance use
The engagement in the use of alcohol, illicit drugs, and cigarette in the past three months will be assessed using the NIDA-Modified Alcohol, Smoking and Substance Involvement Screening (NIDA-ASSIST) [59]. The scale includes 12 dichotomous items (0 = No, 1 = Yes) asking if a participant has engaged in using or misusing any of 12 individual substances. "

6a-i) Online questionnaires: describe if they were validated for online use and apply CHERRIES items to describe how the questionnaires were designed/deployed

If outcomes were obtained through online questionnaires, describe if they were validated for online use and apply CHERRIES items to describe how the questionnaires were designed/deployed [9].

	1	2	3	4	5	
subitem not at all important	○	○	○	○	○	essential

Does your paper address subitem 6a-i?

Copy and paste relevant sections from manuscript text

Your answer

6a-ii) Describe whether and how "use" (including intensity of use/dosage) was defined/measured/monitored

Describe whether and how "use" (including intensity of use/dosage) was defined/measured/monitored (logins, logfile analysis, etc.). Use/adoption metrics are important process outcomes that should be reported in any ehealth trial.

	1	2	3	4	5	
subitem not at all important	○	○	○	○	○	essential

Does your paper address subitem 6a-ii?

Copy and paste relevant sections from manuscript text

Your answer

6a-iii) Describe whether, how, and when qualitative feedback from participants was obtained

Describe whether, how, and when qualitative feedback from participants was obtained (e.g., through emails, feedback forms, interviews, focus groups).

	1	2	3	4	5	
subitem not at all important	○	○	○	○	○	essential

Does your paper address subitem 6a-iii?

Copy and paste relevant sections from manuscript text

Your answer

6b) Any changes to trial outcomes after the trial commenced, with reasons

Does your paper address CONSORT subitem 6b? *

Copy and paste relevant sections from the manuscript (include quotes in quotation marks "like this" to indicate direct quotes from your manuscript), or elaborate on this item by providing additional information not in the ms, or briefly explain why the item is not applicable/relevant for your study

No changes were made after the trial commenced.

7a) How sample size was determined

NPT: When applicable, details of whether and how the clustering by care provides or centers was addressed

7a-i) Describe whether and how expected attrition was taken into account when calculating the sample size

Describe whether and how expected attrition was taken into account when calculating the sample size.

	1	2	3	4	5	
subitem not at all important	○	○	○	○	○	essential

Does your paper address subitem 7a-i?

Copy and paste relevant sections from manuscript title (include quotes in quotation marks "like this" to indicate direct quotes from your manuscript), or elaborate on this item by providing additional information not in the ms, or briefly explain why the item is not applicable/relevant for your study

Your answer

7b) When applicable, explanation of any interim analyses and stopping guidelines

Does your paper address CONSORT subitem 7b? *

Copy and paste relevant sections from the manuscript (include quotes in quotation marks "like this" to indicate direct quotes from your manuscript), or elaborate on this item by providing additional information not in the ms, or briefly explain why the item is not applicable/relevant for your study

N/A: This is the feasibility research of a developmental intervention research. However, peer leaders will be responsible for reporting adverse events to the PI, and the PI, as well as the clinicians, will consider if the relevant events require to discontinue the intervention and provide referrals for psychological services.

8a) Method used to generate the random allocation sequence

NPT: When applicable, how care providers were allocated to each trial group

Does your paper address CONSORT subitem 8a? *

Copy and paste relevant sections from the manuscript (include quotes in quotation marks "like this" to indicate direct quotes from your manuscript), or elaborate on this item by providing additional information not in the ms, or briefly explain why the item is not applicable/relevant for your study

"The intervention modules will be tested through a RCT design using mixed methods in 70 young adults who engage in NMUPO [93]. The primary purpose is to determine acceptability, usability, feasibility, and initial efficacy. Participants reached by peer leaders/ads on social media will be navigated to a screening survey on RedCap and scheduling a meeting with the research team for inform consent. Participants will be required to comply with User Safety Agreement. Participants will be randomly assigned to the parallel intervention (n = 35) or control group (n = 35) using computer-generated random numbers. Participants are blinded to the randomized assignments. "

8b) Type of randomisation; details of any restriction (such as blocking and block size)

Does your paper address CONSORT subitem 8b? *

Copy and paste relevant sections from the manuscript (include quotes in quotation marks "like this" to indicate direct quotes from your manuscript), or elaborate on this item by providing additional information not in the ms, or briefly explain why the item is not applicable/relevant for your study

"The intervention modules will be tested through a RCT design using mixed methods in 70 young adults who engage in NMUPO [93]. The primary purpose is to determine acceptability, usability, feasibility, and initial efficacy. Participants reached by peer leaders/ads on social media will be navigated to a screening survey on RedCap and scheduling a meeting with the research team for inform consent. Participants will be required to comply with User Safety Agreement. Participants will be randomly assigned to the parallel intervention (n = 35) or control group (n = 35) using computer-generated random numbers. Participants are blinded to the randomized assignments. "

9) Mechanism used to implement the random allocation sequence (such as sequentially numbered containers), describing any steps taken to conceal the sequence until interventions were assigned

Does your paper address CONSORT subitem 9? *

Copy and paste relevant sections from the manuscript (include quotes in quotation marks "like this" to indicate direct quotes from your manuscript), or elaborate on this item by providing additional information not in the ms, or briefly explain why the item is not applicable/relevant for your study

"The intervention modules will be tested through a RCT design using mixed methods in 70 young adults who engage in NMUPO [93]. The primary purpose is to determine acceptability, usability, feasibility, and initial efficacy. Participants reached by peer leaders/ads on social media will be navigated to a screening survey on RedCap and scheduling a meeting with the research team for inform consent. Participants will be required to comply with User Safety Agreement. Participants will be randomly assigned to the parallel intervention (n = 35) or control group (n = 35) using computer-generated random numbers. Participants are blinded to the randomized assignments. "

10) Who generated the random allocation sequence, who enrolled participants, and who assigned participants to interventions

Does your paper address CONSORT subitem 10? *

Copy and paste relevant sections from the manuscript (include quotes in quotation marks "like this" to indicate direct quotes from your manuscript), or elaborate on this item by providing additional information not in the ms, or briefly explain why the item is not applicable/relevant for your study

"The intervention modules will be tested through a RCT design using mixed methods in 70 young adults who engage in NMUPO [93]. The primary purpose is to determine acceptability, usability, feasibility, and initial efficacy. Participants reached by peer leaders/ads on social media will be navigated to a screening survey on RedCap and scheduling a meeting with the research team for inform consent. Participants will be required to comply with User Safety Agreement. Participants will be randomly assigned to the parallel intervention (n = 35) or control group (n = 35) using computer-generated random numbers. Participants are blinded to the randomized assignments. "

11a) If done, who was blinded after assignment to interventions (for example, participants, care providers, those assessing outcomes) and how

NPT: Whether or not administering co-interventions were blinded to group assignment

11a-i) Specify who was blinded, and who wasn't

Specify who was blinded, and who wasn't. Usually, in web-based trials it is not possible to blind the participants [1, 3] (this should be clearly acknowledged), but it may be possible to blind outcome assessors, those doing data analysis or those administering co-interventions (if any).

	1	2	3	4	5	
subitem not at all important	○	○	○	○	○	essential

Does your paper address subitem 11a-i? *

Copy and paste relevant sections from the manuscript (include quotes in quotation marks "like this" to indicate direct quotes from your manuscript), or elaborate on this item by providing additional information not in the ms, or briefly explain why the item is not applicable/relevant for your study

"The intervention modules will be tested through a RCT design using mixed methods in 70 young adults who engage in NMUPO [93]. The primary purpose is to determine acceptability, usability, feasibility, and initial efficacy. Participants reached by peer leaders/ads on social media will be navigated to a screening survey on RedCap and scheduling a meeting with the research team for inform consent. Participants will be required to comply with User Safety Agreement. Participants will be randomly assigned to the parallel intervention (n = 35) or control group (n = 35) using computer-generated random numbers. Participants are blinded to the randomized assignments. "

11a-ii) Discuss e.g., whether participants knew which intervention was the "intervention of interest" and which one was the "comparator"

Informed consent procedures (4a-ii) can create biases and certain expectations - discuss e.g., whether participants knew which intervention was the "intervention of interest" and which one was the "comparator".

	1	2	3	4	5	
subitem not at all important	○	○	○	○	○	essential

Does your paper address subitem 11a-ii?

Copy and paste relevant sections from the manuscript (include quotes in quotation marks "like this" to indicate direct quotes from your manuscript), or elaborate on this item by providing additional information not in the ms, or briefly explain why the item is not applicable/relevant for your study

Your answer

11b) If relevant, description of the similarity of interventions

(this item is usually not relevant for ehealth trials as it refers to similarity of a placebo or sham intervention to a active medication/intervention)

Does your paper address CONSORT subitem 11b? *

Copy and paste relevant sections from the manuscript (include quotes in quotation marks "like this" to indicate direct quotes from your manuscript), or elaborate on this item by providing additional information not in the ms, or briefly explain why the item is not applicable/relevant for your study

N/A: The control group will not receive any intervention.

12a) Statistical methods used to compare groups for primary and secondary outcomes

NPT: When applicable, details of whether and how the clustering by care providers or centers was addressed

Does your paper address CONSORT subitem 12a? *

Copy and paste relevant sections from the manuscript (include quotes in quotation marks "like this" to indicate direct quotes from your manuscript), or elaborate on this item by providing additional information not in the ms, or briefly explain why the item is not applicable/relevant for your study

"Qualitative analyses
Post-intervention interviews will be audio-recorded, transcribed, and coded using a pragmatic approach. The framework analysis [63] will be performed via a deductive process including five key steps: (1) familiarizing the data; (2) developing a coding scheme; (3) condensing and structuring the data; (4) rearranging the coded data and comparing patterns; (5) mapping and interpretation [64]. A codebook will be developed based on step (2) as a guide for coding. Coding disagreements will be resolved through group discussion. Data analysis will be conducted by the PI and graduate assistants on NVivo 11.0. Interrater reliability will be assessed throughout the coding process with a goal of >.80.[65] Analyses for post-intervention interviews will synthesize the comments on feasibility, acceptability, and usability of the intervention, as well as summarize suggestions for improvements.
Quantitative analyses
Bivariate analyses
Data of web-based surveys (i.e., pre- and post-intervention) will be screened for missing, outliers, and normality. If data are not missing (completely) at random or have high missing rate (>5%), multiple imputation will be employed [107]. Transformations will be conducted if violating normality (square-root for moderate skew or log for greater skew). Attrition tests will be employed (i.e., chi-square tests and analysis of variance) on demographics and outcomes at baseline. Descriptive statistics will be reported on primary (feasibility outcomes) and secondary outcomes (psychosocial and behavioral outcomes).
Multivariate analyses
Preliminary outcomes from baseline and 12-week will be tested using repeated measures Analysis of Covariance (ANCOVA), controlling for demographics. EMA data will be tested using multilevel modeling (i.e., random-coefficient regression model) to examine intervention usability and acceptability, capable for incorporating tests of time-serial characteristics including linear trend over time and cyclicity. Prior to multilevel modeling, unconditional means models will be tested for between- and within-subjects variation in each measure and determination of appropriate within-person error covariance structure. [108]
Mixed method analysis
Qualitative and quantitative data on feasibility will be triangulated with a complementary proposition, [109]in which the qualitative findings will provide in-dept understanding for the quantitative results in terms of intervention feasibility outcomes."

12a-i) Imputation techniques to deal with attrition / missing values

Imputation techniques to deal with attrition / missing values: Not all participants will use the intervention/comparator as intended and attrition is typically high in ehealth trials. Specify how participants who did not use the application or dropped out from the trial were treated in the statistical analysis (a complete case analysis is strongly discouraged, and simple imputation techniques such as LOCF may also be problematic [4]).

	1	2	3	4	5	
subitem not at all important	○	○	○	○	○	essential

Does your paper address subitem 12a-i? *

Copy and paste relevant sections from the manuscript (include quotes in quotation marks "like this" to indicate direct quotes from your manuscript), or elaborate on this item by providing additional information not in the ms, or briefly explain why the item is not applicable/relevant for your study

N/A: The primary purpose of the study is feasibility assessment.

12b) Methods for additional analyses, such as subgroup analyses and adjusted analyses

Does your paper address CONSORT subitem 12b? *

Copy and paste relevant sections from the manuscript (include quotes in quotation marks "like this" to indicate direct quotes from your manuscript), or elaborate on this item by providing additional information not in the ms, or briefly explain why the item is not applicable/relevant for your study

N/A: The primary purpose of the study is feasibility assessment.

X26) REB/IRB Approval and Ethical Considerations [recommended as subheading under "Methods"] (not a CONSORT item)

X26-i) Comment on ethics committee approval

	1	2	3	4	5	
subitem not at all important	○	○	○	○	○	essential

Does your paper address subitem X26-i?

Copy and paste relevant sections from the manuscript (include quotes in quotation marks "like this" to indicate direct quotes from your manuscript), or elaborate on this item by providing additional information not in the ms, or briefly explain why the item is not applicable/relevant for your study

Your answer

x26-ii) Outline informed consent procedures

Outline informed consent procedures e.g., if consent was obtained offline or online (how? Checkbox, etc.?), and what information was provided (see 4a-ii). See [6] for some items to be included in informed consent documents.

	1	2	3	4	5	
subitem not at all important	○	○	○	○	○	essential

Does your paper address subitem X26-ii?

Copy and paste relevant sections from the manuscript (include quotes in quotation marks "like this" to indicate direct quotes from your manuscript), or elaborate on this item by providing additional information not in the ms, or briefly explain why the item is not applicable/relevant for your study

Your answer

X26-iii) Safety and security procedures

Safety and security procedures, incl. privacy considerations, and any steps taken to reduce the likelihood or detection of harm (e.g., education and training, availability of a hotline)

	1	2	3	4	5	
subitem not at all important	○	○	○	○	○	essential

Does your paper address subitem X26-iii?

Copy and paste relevant sections from the manuscript (include quotes in quotation marks "like this" to indicate direct quotes from your manuscript), or elaborate on this item by providing additional information not in the ms, or briefly explain why the item is not applicable/relevant for your study

Your answer

RESULTS

13a) For each group, the numbers of participants who were randomly assigned, received intended treatment, and were analysed for the primary outcome

NPT: The number of care providers or centers performing the intervention in each group and the number of patients treated by each care provider in each center

Does your paper address CONSORT subitem 13a? *

Copy and paste relevant sections from the manuscript (include quotes in quotation marks "like this" to indicate direct quotes from your manuscript), or elaborate on this item by providing additional information not in the ms, or briefly explain why the item is not applicable/relevant for your study

N/A: By the time of the submission, the proposed study is at the formative study phase (Aim 1), which has not yet involved RCT assignments. However, the recruitment information of the formative interviews have reported in the result section.

13b) For each group, losses and exclusions after randomisation, together with reasons

Does your paper address CONSORT subitem 13b? (NOTE: Preferably, this is shown in a CONSORT flow diagram) *

Copy and paste relevant sections from the manuscript (include quotes in quotation marks "like this" to indicate direct quotes from your manuscript), or elaborate on this item by providing additional information not in the ms, or briefly explain why the item is not applicable/relevant for your study

N/A: By the time of the submission, the proposed study is at the formative study phase (Aim 1), which has not yet involved RCT assignments. However, the recruitment information of the formative interviews have reported in the result section (CONSORT flow chart).

13b-i) Attrition diagram

Strongly recommended: An attrition diagram (e.g., proportion of participants still logging in or using the intervention/comparator in each group plotted over time, similar to a survival curve) or other figures or tables demonstrating usage/dose/engagement.

	1	2	3	4	5	
subitem not at all important	○	○	○	○	○	essential

Does your paper address subitem 13b-i?

Copy and paste relevant sections from the manuscript or cite the figure number if applicable (include quotes in quotation marks "like this" to indicate direct quotes from your manuscript), or elaborate on this item by providing additional information not in the ms, or briefly explain why the item is not applicable/relevant for your study

Your answer

14a) Dates defining the periods of recruitment and follow-up

Does your paper address CONSORT subitem 14a? *

Copy and paste relevant sections from the manuscript (include quotes in quotation marks "like this" to indicate direct quotes from your manuscript), or elaborate on this item by providing additional information not in the ms, or briefly explain why the item is not applicable/relevant for your study

N/A: By the time of the submission, the proposed study is at the formative study phase (Aim 1), which has not yet involved RCT assignments. However, the recruitment information of the formative interviews have reported in the result section (CONSORT flow chart).

14a-i) Indicate if critical "secular events" fell into the study period

Indicate if critical "secular events" fell into the study period, e.g., significant changes in Internet resources available or "changes in computer hardware or Internet delivery resources"

	1	2	3	4	5	
subitem not at all important	○	○	○	○	○	essential

Does your paper address subitem 14a-i?

Copy and paste relevant sections from the manuscript (include quotes in quotation marks "like this" to indicate direct quotes from your manuscript), or elaborate on this item by providing additional information not in the ms, or briefly explain why the item is not applicable/relevant for your study

Your answer

14b) Why the trial ended or was stopped (early)

Does your paper address CONSORT subitem 14b? *

Copy and paste relevant sections from the manuscript (include quotes in quotation marks "like this" to indicate direct quotes from your manuscript), or elaborate on this item by providing additional information not in the ms, or briefly explain why the item is not applicable/relevant for your study

N/A. N/A: By the time of the submission, the proposed study is at the formative study phase (Aim 1), which has not yet involved RCT assignments. However, the recruitment information of the formative interviews have reported in the result section (CONSORT flow chart).

15) A table showing baseline demographic and clinical characteristics for each group

NPT: When applicable, a description of care providers (case volume, qualification, expertise, etc.) and centers (volume) in each group

Does your paper address CONSORT subitem 15? *

Copy and paste relevant sections from the manuscript (include quotes in quotation marks "like this" to indicate direct quotes from your manuscript), or elaborate on this item by providing additional information not in the ms, or briefly explain why the item is not applicable/relevant for your study

N/A: By the time of the submission, the proposed study is at the formative study phase (Aim 1), which has not yet involved RCT assignments. However, the recruitment information of the formative interviews have reported in the result section (CONSORT flow chart).

15-i) Report demographics associated with digital divide issues

In ehealth trials it is particularly important to report demographics associated with digital divide issues, such as age, education, gender, social-economic status, computer/Internet/ehealth literacy of the participants, if known.

	1	2	3	4	5	
subitem not at all important	○	○	○	○	○	essential

Does your paper address subitem 15-i? *

Copy and paste relevant sections from the manuscript (include quotes in quotation marks "like this" to indicate direct quotes from your manuscript), or elaborate on this item by providing additional information not in the ms, or briefly explain why the item is not applicable/relevant for your study

N/A. The intervention has not yet developed and implemented.

16) For each group, number of participants (denominator) included in each analysis and whether the analysis was by original assigned groups

16-i) Report multiple "denominators" and provide definitions

Report multiple "denominators" and provide definitions: Report N's (and effect sizes) "across a range of study participation [and use] thresholds" [1], e.g., N exposed, N consented, N used more than x times, N used more than y weeks, N participants "used" the intervention/comparator at specific pre-defined time points of interest (in absolute and relative numbers per group). Always clearly define "use" of the intervention.

	1	2	3	4	5	
subitem not at all important	○	○	○	○	○	essential

Does your paper address subitem 16-i? *

Copy and paste relevant sections from the manuscript (include quotes in quotation marks "like this" to indicate direct quotes from your manuscript), or elaborate on this item by providing additional information not in the ms, or briefly explain why the item is not applicable/relevant for your study

N/A. Intervention has not yet developed and implemented. However, the dose of intervention is a part of the primary outcomes as noted in the measures section.

16-ii) Primary analysis should be intent-to-treat

Primary analysis should be intent-to-treat, secondary analyses could include comparing only "users", with the appropriate caveats that this is no longer a randomized sample (see 18-i).

	1	2	3	4	5	
subitem not at all important	○	○	○	○	○	essential

Does your paper address subitem 16-ii?

Copy and paste relevant sections from the manuscript (include quotes in quotation marks "like this" to indicate direct quotes from your manuscript), or elaborate on this item by providing additional information not in the ms, or briefly explain why the item is not applicable/relevant for your study

Your answer

17a) For each primary and secondary outcome, results for each group, and the estimated effect size and its precision (such as 95% confidence interval)

Does your paper address CONSORT subitem 17a? *

Copy and paste relevant sections from the manuscript (include quotes in quotation marks "like this" to indicate direct quotes from your manuscript), or elaborate on this item by providing additional information not in the ms, or briefly explain why the item is not applicable/relevant for your study

N/A. This is a protocol manuscript.

17a-i) Presentation of process outcomes such as metrics of use and intensity of use

In addition to primary/secondary (clinical) outcomes, the presentation of process outcomes such as metrics of use and intensity of use (dose, exposure) and their operational definitions is critical. This does not only refer to metrics of attrition (13-b) (often a binary variable), but also to more continuous exposure metrics such as "average session length". These must be accompanied by a technical description how a metric like a "session" is defined (e.g., timeout after idle time) [1] (report under item 6a).

	1	2	3	4	5	
subitem not at all important	○	○	○	○	○	essential

Does your paper address subitem 17a-i?

Copy and paste relevant sections from the manuscript (include quotes in quotation marks "like this" to indicate direct quotes from your manuscript), or elaborate on this item by providing additional information not in the ms, or briefly explain why the item is not applicable/relevant for your study

Your answer

17b) For binary outcomes, presentation of both absolute and relative effect sizes is recommended

Does your paper address CONSORT subitem 17b? *

Copy and paste relevant sections from the manuscript (include quotes in quotation marks "like this" to indicate direct quotes from your manuscript), or elaborate on this item by providing additional information not in the ms, or briefly explain why the item is not applicable/relevant for your study

N/A. This is a protocol manuscript.

18) Results of any other analyses performed, including subgroup analyses and adjusted analyses, distinguishing pre-specified from exploratory

Does your paper address CONSORT subitem 18? *

Copy and paste relevant sections from the manuscript (include quotes in quotation marks "like this" to indicate direct quotes from your manuscript), or elaborate on this item by providing additional information not in the ms, or briefly explain why the item is not applicable/relevant for your study

N/A. This is a protocol manuscript.

18-i) Subgroup analysis of comparing only users

A subgroup analysis of comparing only users is not uncommon in ehealth trials, but if done, it must be stressed that this is a self-selected sample and no longer an unbiased sample from a randomized trial (see 16-iii).

	1	2	3	4	5	
subitem not at all important	○	○	○	○	○	essential

Does your paper address subitem 18-i?

Copy and paste relevant sections from the manuscript (include quotes in quotation marks "like this" to indicate direct quotes from your manuscript), or elaborate on this item by providing additional information not in the ms, or briefly explain why the item is not applicable/relevant for your study

Your answer

19) All important harms or unintended effects in each group

(for specific guidance see CONSORT for harms)

Does your paper address CONSORT subitem 19? *

Copy and paste relevant sections from the manuscript (include quotes in quotation marks "like this" to indicate direct quotes from your manuscript), or elaborate on this item by providing additional information not in the ms, or briefly explain why the item is not applicable/relevant for your study

N/A. However, peer leaders will be trained and responsible for reporting adverse events to the PI, and the PI, as well as the clinicians, will consider if the relevant events require to discontinue the intervention and provide referrals for psychological services.

19-i) Include privacy breaches, technical problems

Include privacy breaches, technical problems. This does not only include physical "harm" to participants, but also incidents such as perceived or real privacy breaches [1], technical problems, and other unexpected/unintended incidents. "Unintended effects" also includes unintended positive effects [2].

	1	2	3	4	5	
subitem not at all important	○	○	○	○	○	essential

Does your paper address subitem 19-i?

Copy and paste relevant sections from the manuscript (include quotes in quotation marks "like this" to indicate direct quotes from your manuscript), or elaborate on this item by providing additional information not in the ms, or briefly explain why the item is not applicable/relevant for your study

Your answer

19-ii) Include qualitative feedback from participants or observations from staff/researchers

Include qualitative feedback from participants or observations from staff/researchers, if available, on strengths and shortcomings of the application, especially if they point to unintended/unexpected effects or uses. This includes (if available) reasons for why people did or did not use the application as intended by the developers.

	1	2	3	4	5	
subitem not at all important	○	○	○	○	○	essential

Does your paper address subitem 19-ii?

Copy and paste relevant sections from the manuscript (include quotes in quotation marks "like this" to indicate direct quotes from your manuscript), or elaborate on this item by providing additional information not in the ms, or briefly explain why the item is not applicable/relevant for your study

Your answer

DISCUSSION

22) Interpretation consistent with results, balancing benefits and harms, and considering other relevant evidence

NPT: In addition, take into account the choice of the comparator, lack of or partial blinding, and unequal expertise of care providers or centers in each group

22-i) Restate study questions and summarize the answers suggested by the data, starting with primary outcomes and process outcomes (use)

Restate study questions and summarize the answers suggested by the data, starting with primary outcomes and process outcomes (use).

	1	2	3	4	5	
subitem not at all important	○	○	○	○	○	essential

Does your paper address subitem 22-i? *

Copy and paste relevant sections from the manuscript (include quotes in quotation marks "like this" to indicate direct quotes from your manuscript), or elaborate on this item by providing additional information not in the ms, or briefly explain why the item is not applicable/relevant for your study

"In line with previous intervention studies for NMUPO on social media [45], we anticipate that our intervention will yield high feasibility outcomes. In particular, the mixed method assessments for the intervention group would demonstrate that (1) high feasibility, acceptability, and usability on all intervention modules among young adults across 12 weeks; (2) high engagements especially for the modules in the interactive formats, such as discuss polls and living streaming group meetings. We also anticipate a significant preliminary intervention efficacy on the NMUPO-related and psychosocial behavior outcomes. Compared to the control group, intervention would report (1) decreases of past-three-month NMUPO and other substance use (e.g., alcohol, illicit drugs, and cigarette); (2) decreases of psychological distress; (3) changes of outcome expectancies (e.g., increased physical discomfort and decreased pain reduction); (4) increases of NMUPO knowledge and NMUPO risk perception; and (5) increases of resilience and self-efficacy (i.e., action and coping). "

22-ii) Highlight unanswered new questions, suggest future research

Highlight unanswered new questions, suggest future research.

	1	2	3	4	5	
subitem not at all important	○	○	○	○	○	essential

Does your paper address subitem 22-ii?

Copy and paste relevant sections from the manuscript (include quotes in quotation marks "like this" to indicate direct quotes from your manuscript), or elaborate on this item by providing additional information not in the ms, or briefly explain why the item is not applicable/relevant for your study

Your answer

20) Trial limitations, addressing sources of potential bias, imprecision, and, if relevant, multiplicity of analyses

20-i) Typical limitations in ehealth trials

Typical limitations in ehealth trials: Participants in ehealth trials are rarely blinded. Ehealth trials often look at a multiplicity of outcomes, increasing risk for a Type I error. Discuss biases due to non-use of the intervention/usability issues, biases through informed consent procedures, unexpected events.

	1	2	3	4	5	
subitem not at all important	○	○	○	○	○	essential

Does your paper address subitem 20-i? *

Copy and paste relevant sections from the manuscript (include quotes in quotation marks "like this" to indicate direct quotes from your manuscript), or elaborate on this item by providing additional information not in the ms, or briefly explain why the item is not applicable/relevant for your study

"The proposed study encounters several methodological limitations. First, the generalization of our findings will be limited by the convenient sampling approach. Second, since most evaluations in this study will be based on self-reported measures, results would be subject to response bias (e.g., social desirability and recall bias). Third, our EMAs would impose additional burdens on participants due to the frequent prompts for assessments at real-time. Fourth, our efficacy findings would be limited in assessing long-term effects given a pre- and post-evaluation design. Fifth, since the planned contents for all peer-led groups will be identical and delivered concurrently, our analyses could not examine the efficacy of specific intervention modules. Sixth, given a program will be delivered on Instagram, our findings would be limited to Instagram users, and the format and presentation of the intervention contents would be constrained by the platform's available features and functionality. Seventh, our peer leaders would also have limitations. They are recruited solely from local agencies within a single state (South Carolina). In addition, although they will be supported by clinicians (e.g., licensed clinical psychologists and physicians in addiction medicine), their service may be subject to disparities in the quality of support. To address those, our future plan is to conduct a scaled-up cross-platform social media RCT study with a design of the Multiphase Optimization Strategy and longitudinal measures. The intervention will be provided by a team of peer leaders, comprising recovery coaches with expertise in opioid misuse from various states across the United States."

21) Generalisability (external validity, applicability) of the trial findings

NPT: External validity of the trial findings according to the intervention, comparators, patients, and care providers or centers involved in the trial

21-i) Generalizability to other populations

Generalizability to other populations: In particular, discuss generalizability to a general Internet population, outside of a RCT setting, and general patient population, including applicability of the study results for other organizations

	1	2	3	4	5	
subitem not at all important	○	○	○	○	○	essential

Does your paper address subitem 21-i?

Copy and paste relevant sections from the manuscript (include quotes in quotation marks "like this" to indicate direct quotes from your manuscript), or elaborate on this item by providing additional information not in the ms, or briefly explain why the item is not applicable/relevant for your study

Your answer

21-ii) Discuss if there were elements in the RCT that would be different in a routine application setting

Discuss if there were elements in the RCT that would be different in a routine application setting (e.g., prompts/reminders, more human involvement, training sessions or other co-interventions) and what impact the omission of these elements could have on use, adoption, or outcomes if the intervention is applied outside of a RCT setting.

	1	2	3	4	5	
subitem not at all important	○	○	○	○	○	essential

Does your paper address subitem 21-ii?

Copy and paste relevant sections from the manuscript (include quotes in quotation marks "like this" to indicate direct quotes from your manuscript), or elaborate on this item by providing additional information not in the ms, or briefly explain why the item is not applicable/relevant for your study

Your answer

OTHER INFORMATION

23) Registration number and name of trial registry

Does your paper address CONSORT subitem 23? *

Copy and paste relevant sections from the manuscript (include quotes in quotation marks "like this" to indicate direct quotes from your manuscript), or elaborate on this item by providing additional information not in the ms, or briefly explain why the item is not applicable/relevant for your study

"This trial is registered at ClincialTrials.gov, registration number: NCT06469749, registered on June 25, 2024 "

24) Where the full trial protocol can be accessed, if available

Does your paper address CONSORT subitem 24? *

Cite a Multimedia Appendix, other reference, or copy and paste relevant sections from the manuscript (include quotes in quotation marks "like this" to indicate direct quotes from your manuscript), or elaborate on this item by providing additional information not in the ms, or briefly explain why the item is not applicable/relevant for your study

N/A: If this manuscript is published, the full trial protocol will be available to public.

25) Sources of funding and other support (such as supply of drugs), role of funders

Does your paper address CONSORT subitem 25? *

Copy and paste relevant sections from the manuscript (include quotes in quotation marks "like this" to indicate direct quotes from your manuscript), or elaborate on this item by providing additional information not in the ms, or briefly explain why the item is not applicable/relevant for your study

"This protocol reported in this manuscript was supported by the National Institute on Drug Abuse (Award number: 1K01DA058768-01). The content solely reflects the responsibility of the authors and does not necessarily represent the official views of the National Institutes of Health."

X27) Conflicts of Interest (not a CONSORT item)

X27-i) State the relation of the study team towards the system being evaluated

In addition to the usual declaration of interests (financial or otherwise), also state the relation of the study team towards the system being evaluated, i.e., state if the authors/evaluators are distinct from or identical with the developers/sponsors of the intervention.

	1	2	3	4	5	
subitem not at all important	○	○	○	○	○	essential

Does your paper address subitem X27-i?

Copy and paste relevant sections from the manuscript (include quotes in quotation marks "like this" to indicate direct quotes from your manuscript), or elaborate on this item by providing additional information not in the ms, or briefly explain why the item is not applicable/relevant for your study

Your answer

About the CONSORT EHEALTH checklist

As a result of using this checklist, did you make changes in your manuscript? *

- ○ yes, major changes
- ◉ yes, minor changes
- ○ no

What were the most important changes you made as a result of using this checklist?

Your answer

How much time did you spend on going through the checklist INCLUDING making changes in your manuscript *

It took about one month to go through the checklist and make changes.

As a result of using this checklist, do you think your manuscript has improved? *

- ◉ yes
- ○ no
- ○ Other:

Would you like to become involved in the CONSORT EHEALTH group?

This would involve for example becoming involved in participating in a workshop and writing an "Explanation and Elaboration" document

- ○ yes
- ○ no
- ○ Other:

Any other comments or questions on CONSORT EHEALTH

Your answer

STOP - Save this form as PDF before you click submit

To generate a record that you filled in this form, we recommend to generate a PDF of this page (on a Mac, simply select "print" and then select "print as PDF") before you submit it.

When you submit your (revised) paper to JMIR, please upload the PDF as supplementary file.

Don't worry if some text in the textboxes is cut off, as we still have the complete information in our database. Thank you!

Final step: Click submit !

Click submit so we have your answers in our database!

Submit Clear form

Never submit passwords through Google Forms.

This content is neither created nor endorsed by Google. - Terms of Service - Privacy Policy

Does this form look suspicious? Report

Your response is too large. Try shortening some answers.
